# Short-Term In-Vitro Expansion Improves Monitoring and Allows Affordable Generation of Virus-Specific T-Cells against Several Viruses for a Broad Clinical Application

**DOI:** 10.1371/journal.pone.0059592

**Published:** 2013-04-22

**Authors:** René Geyeregger, Christine Freimüller, Stefan Stevanovic, Julia Stemberger, Gabor Mester, Jasmin Dmytrus, Thomas Lion, Hans-Georg Rammensee, Gottfried Fischer, Britta Eiz-Vesper, Anita Lawitschka, Susanne Matthes, Gerhard Fritsch

**Affiliations:** 1 Department of Clinical Cell Biology and FACS Core Unit, Children's Cancer Research Institute (CCRI), Vienna, Austria; 2 Department Pediatrics, Medical University of Vienna, Vienna, Austria; 3 Department of Molecular Microbiology, Children's Cancer Research Institute (CCRI), Vienna, Austria; 4 Department of Immunology, Institute for Cell Biology, Eberhard-Karls-Universität Tübingen, Tübingen, Germany; 5 Department of Blood Group Serology and Transfusion Medicine, Medical University of Vienna, Vienna, Austria; 6 Institute for Transfusion Medicine, Hannover Medical School, Hannover, Germany; 7 Department of Stem Cell Transplantation, St. Anna Children's Hospital, Vienna, Austria; Baylor College of Medicine, United States of America

## Abstract

Adenoviral infections are a major cause of morbidity and mortality after allogeneic hematopoietic stem cell transplantation (HSCT) in pediatric patients. Adoptive transfer of donor-derived human adenovirus (HAdV)-specific T-cells represents a promising treatment option. However, the difficulty in identifying and selecting rare HAdV-specific T-cells, and the short time span between patients at high risk for invasive infection and viremia are major limitations. We therefore developed an IL-15-driven 6 to 12 day short-term protocol for *in vitro* detection of HAdV-specific T cells, as revealed by known MHC class I multimers and a newly identified adenoviral CD8 T-cell epitope derived from the E1A protein for the frequent HLA-type A*02∶01 and IFN-γ. Using this novel and improved diagnostic approach we observed a correlation between adenoviral load and reconstitution of CD8^+^ and CD4^+^ HAdV-specific T-cells including central memory cells in HSCT-patients. Adaption of the 12-day protocol to good manufacturing practice conditions resulted in a 2.6-log (mean) expansion of HAdV-specific T-cells displaying high cytolytic activity (4-fold) compared to controls and low or absent alloreactivity. Similar protocols successfully identified and rapidly expanded CMV-, EBV-, and BKV-specific T-cells. Our approach provides a powerful clinical-grade convertible tool for rapid and cost-effective detection and enrichment of multiple virus-specific T-cells that may facilitate broad clinical application.

## Introduction

Adenovirus (HAdV), cytomegalovirus (CMV), Epstein-Barr-Virus (EBV), and polyoma-Virus (BKV) are responsible for serious morbidity and mortality in patients after hematopoietic stem cell transplantation (HSCT) [1], [2], [3], [4]. HAdV represents one of the most frequent and dangerous infections post transplant [5], [6], especially after haploidentical HSCT [1], [5], [7] and, therefore, is a front-ranking target for early preemptive antiviral therapy [8]. Unfortunately, prophylactic treatment with anti-viral drugs is of limited effectiveness, expensive and associated with substantial toxicity, and may result in overtreatment of patients [1], [6], [9], [10]. Recently, it has been shown that reconstitution of HAdV-specific T-cell response correlates with clearance of ADV infection [11], [12], [13], [14]. In patients who showed no virus-specific immune reconstitution after HSCT, donor-derived virus-specific T-cells against different viruses including HAdV were administered with impressive clinical results [15], [16], [17], [18], [19], [20], [21], [22]. As a prerequisite for the monitoring of virus-specific T-cells in donors and patients, immunodominant viral epitopes have to be identified. Altough we focused only on the monitoring of HAdV-specific T cells, new epitopes could also be used for adoptive therapy, i.e. for the magnetic isolation of HAdV-mulitmer+ T cells [22]. Certain sequences of the major capsid protein hexon are highly conserved among human HAdV which currently comprise more than 55 sybtypes divided into 7 different species (A–G)[23]. This provides the basis for “cross-reactivity” of HAdV-specific T-cells facilitating broad recognition and protection against several species [24]. It is known that most CD4^+^ and CD8^+^ ADV-specific T-cells recognize predominantly hexon protein structures or overlapping 15-mer peptide pools. The IFN-γ secretion induced by appropriate stimulation enables their detection by the IFN-γ -cytokine secretion assay (CSA) [25], [26]. Alternatively, virus-specific T-cells can be identified and isolated using different types of MHC class I multimers including tetramers, pentamers or streptamers [24]. To date, only few HAdV-specific immunodominant CD8^+^ T-cell epitopes have been identified that are presented in the context of the common HLA-types A*01, A*24, B*07 and B*35 [14], [27] thus greatly limiting the number of available HAdV-multimers. Using these four multimers, the probability to detect ADV-specific T-cells within the Caucasian population is about 73%. According to an algorithm presented by Schipper et al [28], this percentage could be increased to 95%, if a functional A*02-based multimer were available. Our primary aim was therefore to identify new promising ADV-specific epitopes for the HLA-types A*01 and A*24, and particularly for the frequent HLA-type A*02, by analyzing the main structural proteins of the virus, including hexon and protein II, as well as the E1A protein expressed very early after infection.

The utility of HAdV-specific multimers for diagnostic applications is further supported by the recent observation that, in patients who cleared HAdV-infection after HSCT, apart from CD4^+^-, also CD8^+^ T-cells were present. [14]. However, in most healthy donors and HSCT-patients, HAdV-specific T-cells were reliably detectable only after *in vitro* culture with HAdV-antigen [14], [29]. Due to the low frequency of circulating the HAdV-specific T-cells, their exact phenotype remains to be elucidated.

Current clinical immunotherapy protocols are based on either long-term *in vitro* expansion, excluding [15] or including transfected antigen-presenting cells (APCs) [16], [17]. Alternatively, direct magnetic selection of virus-specific T-cells using the IFN-γ -CSA [18], [20], tetramers [19] or pentamers [22] is employed. More recently good manufacturing practice (GMP)-compliant removable streptamers became available that represent the only therapy presently not considered as an “Advanced Therapy Medicinal Product (ATMP)” [21]. Although all studies referenced above reported prevention of overt viral disease and only mild or no graft versus host disease (GvHD), they have a number of important limitations: some are very time-consuming (10–14 weeks), technically demanding and cost intensive, others involve gene therapy, require large volumes of blood, or are limited to those patients that express HLA alleles for which multimers are available. These constraints represent a major impediment to broad clinical application of these adoptive immunotherapy approaches [30]. The first attempt to use only synthetic peptide mixes and cytokines to rapidly generate virus-specific T-cells within 9–16 days was recently published [31]. A major focus of this study was to evaluate optimal conditions for T-cell expansion by testing different viral peptide concentrations and cytokines (preferential IL-4 and IL-7). However, relevant cytolytic activity (<10%) of e.g. expanded HAdV-specific T-cells was only shown after 16 days of expansion. In our study, fresh/frozen PBMCs were stimulated twice within only 12 days by using GMP-compliant adaptable peptide mixes and a consciously delayed supplementation of IL-15, which resulted in high numbers of functional and cytolytically active virus-specific T-cells against HAdV, CMV, EBV and BKV. In addition, for the first time, no or only low alloreactivity was evaluated very detailed by several different assays to further proof the safety of these cells.

## Materials and Methods

### Epitope prediction

Epitope candidates were predicted using the SYFPEITHI software (www.syfpeithi.de) [32], [33]. Protein sequences were derived from the SwissProt database (www.uniprot.org release 2010_06): P03277 for hexon Ad2, P04133 for hexon Ad5, P03254 for E1A Ad2, P03255 for E1A Ad5, P03280 for pVIII Ad2, and P24930 for pVIII Ad5.

### Peptide synthesis

Peptides were synthesized by standard Fmoc chemistry using an ABI 433A Synthesizer (Applied Biosystems, Darmstadt, Germany), or an Economy Peptides Synthesizer EPS 221 (ABIMED, Langen, Germany). Synthesis products were analyzed by HPLC (Varian Star, Zinsser Analytics, Munich, Germany) and MALDI-TOF (G2025A, Agilent Technologies, Santa Clara, CA) or electron spray ionization-time of flight (Q-TOF I, Micromass, Manchester, UK) mass spectrometry.

### Cells from donors and patients

PBMCs were isolated by standard Ficoll (PAA, Pasching, Austria) gradient separation and used directly or cryopreserved in fetal calf serum (PAA) or 2% Octaplas (OP, Octapharma, Vienna, Austria) with 10% Dimethylsulfoxide (DMSO, CryoSure, Dessau-Tornau, Germany) until further analysis. Monocytes (purity 70 to 95%) were either positively selected by using CD14 MicroBeads (Miltenyi Biotec, Bergisch Gladbach, Germany) according to the manufactureŕs instructions or isolated after adherence to plastic flasks for 2 h at 37°C in AIM-V medium (Invitrogen, Carlsbad, CA) (1% OP) as described [34], depending on the assay used (see below). To obtain Phytohemagglutinine- (PHA-L; Sigma-Aldrich, St Louis, MO) blasts as targets for the cytotoxicity assay, PBMCs (2×10^6^/ml) were cultured for 6 days in AIM-V supplemented with 2% OP, 2 mM L-Glutamine and 25 mM HEPES, designated as AIM−V+, in the presence of PHA (5 µg/ml). In addition, IL-2 (PeproTech, Rocky Hill, NY) (5 ng/ml) was added on day 3.

### Magnetic selection of ADV-specific T-cells from patients

Five to 8×10^6^ PBMCs were stained with HAdV-specific streptamers according to manufactures instructions (IBA Technologies, Göttingen, Germany), incubated with anti-phycoerythrin(PE) MicroBeads (Miltenyi), magnetically selected by MS-columns (Miltenyi) according to manufactureŕs instructions (Miltenyi) and analyzed by flow cytometry.

### HLA typing of blood donors and patients

Low and high resolution HLA typing of healthy blood donors and patients was performed at the Institute of Transfusion Medicine (Tübingen, Germany) or the Department of Blood Group Serology (Vienna, Austria) with the donorś and patientś written consent.

### Virus-specific and control antigens

Peptides used for multimer analyses are shown in the Table S2 in File S1. Peptide pools for EBV (EBNA-3A) and BKV (LT-Ag) were purchased from JPT (JPT Peptide Technologies, Berlin, Germany) and used at a final concentration of 10 µg/ml for stimulation or pulsing of cells. The final concentration of HAdV (subgroup C-derived Hexon AdV5), EBV (EBNA-1, BZLF-1 and LMP-2) and CMV (pp65) PepTivator (Miltenyi) in the cell suspension was 0.6 nmol for each pepide per ml.

### Quantitative real-time-PCR analysis of viruses from patients

Routine HAdV virus screening of patientśs stool and blood samples was performed by real-time quantitative (RQ) PCR. Viral DNA isolation followed by RQ-PCR were done as described [1].

### In vitro expansion of virus-specific T-cells

For ELIspot analysis, IL-2-expanded peptide-specific T-cells were generated as follows: thawed PBMCs were washed and cultured in Iscove's modified Dulbecco's medium (IMDM) (Lonza, Basel, Switzerland) supplemented with 2% heat-inactivated human serum produced in the laboratory (Tübingen), 50 µM β-mercaptoethanol (Roth), 50 U/ml penicillin, 50 µg/ml streptomycin (both Lonza), and 20 µg/ml gentamicin (Cambrex, Baltimore, USA), and stimulated with peptides (1 µg/ml) on day 2. On days 4 and 6, IL-2 (Promokine) was added at 2 ng/ml. PBMCs were analyzed on day 13 by ELIspot.

For the generation of IL-15-based short-term expanded virus-specific T-cells (seVirus-T-cells), fresh or frozen PBMCs were cultured in AIM−V+ and stimulated with the appropriate peptide pools from HAdV (AdV5-PepTivator), EBV (BZLF-1- and EBNA-1-PepTivator), CMV (pp65-Peptivator), or BKV (LT-Ag-pepmix) antigens for 6 days. On day 6, cultured cells were added to 5×10^6^ post-thaw and adherent monocytes and re-stimulated with a peptide pool. In addition, IL-15 (R&D Systems) was added at 5 ng/ml on days 3 and 9. On day 12, seVirus-T-cells were harvested and used for several analyses. To determine cross-reactivity with other strains of adenovirus, PBMCs were stimulated and expanded as described for the seVirus-T-cells, with the exception that different subgroup-specific peptides were used prior to streptamer analysis. For the monitoring of virus-specific T-cells in donors and patients, PBMCs were mostly stimulated for 6 days, without a second re-stimulation step and IL-15 treatment.

### IFN-γ ELIspot assays

**Figure 1:**
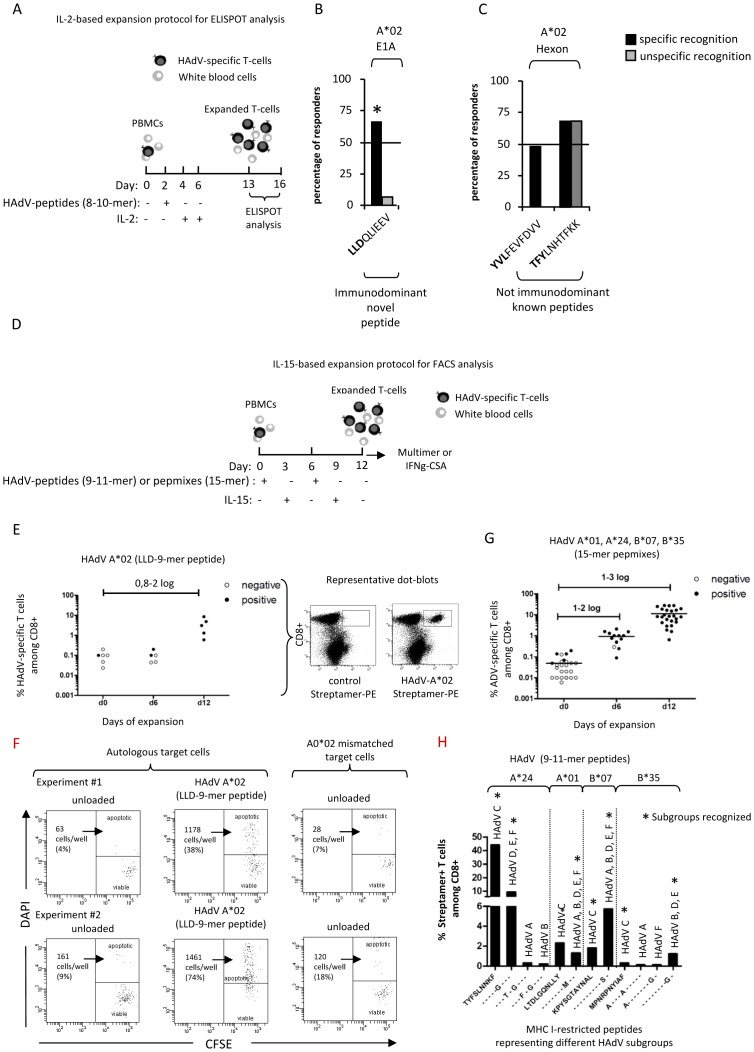
Frequency of responses to novel and known HAdV peptides by ELIspot. Frequency of HAdV-specific T-cells in donors before and after short-term *in vitro* expansion analyzed by multimers and IFNg-CSA. A) Schematic drawing of the IL-2-based *in vitro* expansion protocol for ELIspot analysis. Percentage of donors responding to novel B) and known C) A*02-based peptides by ELIspot assay. Black bars represent donors positive, gray bars those negative for the respective allele. Significant differences are indicated (*, p>0.05, Fisheŕs exact test). D) Schematic drawing of the IL-15-based *in vitro* expansion protocol for FACS analysis. E) Percentages of A*02 ADV-streptamer^+^ T-cells including representative dot plots are given. F) Specific lysis is shown of autologous and allogeneic A*02 mismatched PHA-blasts (target cells), unloaded- or HAdV-A*02 (LLD) peptide-loaded, induced by A*02-multimer-sorted HAdV-T-cells. The total number of “dying” target cells/well and percentage values were evaluated from each sample. Two representative dot plots are shown. G) A*01, A*24, B*07, and B*35 HLA- dependent ADV-specific multimer^+^ (streptamer or pentamer) T-cells among CD8^+^ T-cells, before (day0) and after 6 and 12 days of expansion are shown. Notably, for some donors, multimer analyses were only performed at day 0, 6 and/or 12. H) Subgroup C-derived streptamer staining of PBMCs stimulated with specific peptides for 12 days, representing different adenoviral subgroups, as indicated. The percentage of streptamer^+^ T cells among CD8^+^ is shown. Asterisks represent subgroup recognition.

Five ×10^5^ IL-2 (20 IU/ml)-expanded T-cells/well (see above and Fig. 1A) were seeded and stimulated for 24 h with pools of ADV-derived peptides (1 µg/ml each) or HIV peptide (1 µg/ml) as negative control. As positive control, 10 µg/ml PHA-L (Roche Applied Science, Indianapolis, IN) was used. IFN-γ was detected using the Human IFN-γ ELIspot kit (MabTech, Nacka Strand, Sweden) according to the manufacturer's instructions. Pools eliciting positive responses were split into reactions containing individual peptides and tested again against the respective donor. The cut-off value for a positive response was more than 5 spots per 10^5^ cells exceeding background levels.

### Flow cytometry

PBMCs or seVirus-T-cells were counted on a Sysmex KX21 hematology analyzer (Sysmex, Hyogo, Japan). At least 2.5×10^5^ cells/sample were washed with PBS (PAA), resuspended in 50 µl washing buffer (WB; 0.1% sodium azide, 0.1% BSA in PBS) and incubated with either 7 µl PE-labeled Pentamers (Proimmune, Oxford, UK) or streptamers comprising 1 µl MHC class I and 1.25 µl Strep-Tactin-PE (IBA) for 45 min at 4°C. After two washing steps, cells were resuspended in 50 µl WB, stained with antibodies for 15 min at 4°C, washed again, transferred to Trucount™ tubes (BD Biosciences, San Diego, CA) (optional), and analyzed by flow cytometry. All multimers used are described in Table S2 in File S1. In general, between 95×10^3^ and 500×10^3^ events were acquired. For flow cytometry analyses, the following antibodies were purchases from BD: PE-TR (Texas Red)-labeled anti-CD3 (UCHT1), PerCP-labeled anti-CD3 (SK7), PE-Cy7-labeled anti-CD4 (SK3), Horizon™ V500-labeled anti-CD8 (RPA-T8), APC-Cy7- or PerCP-labeled anti-CD8 (SK1), APC-Cy7-labeled anti-CD19 (SJ25C1), APC-Cy7-labeled anti-CD20 (L27), PE-TR-labeled anti-CD45RA (HI100), Horizon™ V450-labeled anti-CD62L (DREG-56), FITC-labeled anti-CD107a (H4A3), PE-labeled anti-CD137 (1HA2) and FITC-labeled anti-CD56. The PE-labeled anti-CD3 (UCHT1) and PerCP-eFlour® 710-labeled anti-CD4 (SK3) were purchased from DAKO (Glostrup, Denmark) and eBiosciense (San Diego, CA, USA), respectively. For the IFN-γ-CSA, 5×10^5^ cells were washed, resuspended in 100 µl AIM−V+, cultured over night (o/n), and stimulated with the appropriate peptide pools for 4 h. For functional assays, virus-specific T-cell lines (8×10^5^) were mixed at a ratio of 5∶1 with autologous monocytes obtained by CD14 positive selection, and stimulated with the ADV-PepTivator for either 4 h or o/n, depending on whether CD107a or CD137 were analyzed. The subsequent procedure was performed according to the manufactureŕs instructions (Miltenyi). For the intracellular staining of IFN-γ and TNF-α, cells were stimulated for 4 h with the HAdV-peptide pools and stained according to the manufactureŕs instructions (BD Biosciences). Samples without stimulation or stimulated with 1 µg/ml of SEB (Sigma-Aldrich) served as controls.

### General gating strategy and cut-off values

First, beads (if used) were defined. Viable cells were addressed by their appropriate position in the SSC versus FSC plot. Notably, for multimer analysis, CD19^+^ B cells were excluded to avoid false positive results. The analyses were performed either on a FACS LSRII or a LSRFortessa, and the FACSDiva (all BD, Biosciences, CA) was used for data evaluation. The limit of detection for the multimers and the IFN-γ-CSA was defined as >10 positive events and a 5-fold increase compared to the individual negative control.

### CFSE labeling

PBMCs or PHA blasts (10^7^/ml) were resuspended in PBS (0.1% BSA,, Sigma-Aldrich) and labeled with 3 µM CFSE (Sigma-Aldrich) for 10 min at 37°C. The reaction was quenched with 1 ml of Octaplas for 5 min at room temperature (RT). Cells were washed twice with PBS and adjusted to a density of 10^6^ cells/ml in AIM-V+. After incubation o/n at 37°C/5% CO_2_ they were used for proliferation or cytotoxicity assays.

### Proliferation assay

CFSE-labeled PBMCs (2.5×10^6^) were expanded for 12 days according to the IL-15-based *in vitro* expansion protocol described above (Fig. 1D), with the exception of using Cell Proliferation Dye (CPD) eFluor 670 (Ebioscience, San Diego, CA)-labeled monocytes instead of unlabeled monocytes, which enabled exclusion by gating. On day 12, cells were scraped, washed and analyzed by flow cytometry using Trucount™ tubes to determine the percentage and absolute cell number of viable proliferating cells.

### MLR

10^5^ CFSE-labeled PBMCs or seHAdV-T-cells were incubated with 10^5^ autologous or allogeneic 30Gy-irradiated PBMCs in 100 µl AIM−V+ in a 96 well (round bottom) microtiter plate. On day 7, the cell suspension was transferred into Trucount™ tubes and residual alloreactivity, represented by the total number of viable proliferating (CFSE low) cells, was analyzed by flow cytometry.

### Cytotoxicity assay

The cytolytic activity of seVirus-T-cells was assessed by flow cytometry. Notably, only viable cells (>70%), sorted on a FACSAria, were used. CFSE-labeled PHA targets were pulsed with the appropriate viral peptides or peptide pools for 2 hours or o/n, respectively. Unpulsed and control peptide-pulsed targets were used as negative controls. Autologous and allogeneic targets (1.25×10^4^) were mixed with seVirus-T-cells at a ratio 1∶20. Of note, due to low numbers of A*02-sorted HAdV-specific T-cells, only 1.9×10^3^ target cells were used. 4 h after incubation at 37°C, the cell suspension was transferred to Trucount™ tubes and stained with DAPI (0.03 µg/ml). The absolute number of late apoptotic/necrotic targets (CFSE+/DAPI+) was analyzed. Thawed seHAdV-T-cells were cultured for 3 days before cytolytic acitivity was tested.

### Ethics Statement

Cells from donors and patients were obtained upon approval from the local Ethics Committees of Tübingen and Vienna (EK Nr.514/2011) and (EK Nr.024/2011) and informed consent.

### Statistical analysis

Fisher's exact test, p = 0.05 was used for IFN-γ - ELIspot data and student´s t test analysis was employed to determine the statistical significance (P) of all other findings. Data are shown as mean value with standard deviation and/or range.

## Results

### Prediction, identification and characterization of novel and known human ADV-epitopes

In order to identify new adenoviral epitopes for CD8^+^ T-cell responses, we first predicted epitope candidates for the frequent HLA-types A*02, A*01, and A*24 using the SYFPEITHI software (www.syfpeithi.de). We chose two widespread adenoviral strains, Ad2 and Ad5, and focused on three proteins: protein II (hexon, major capsid protein), protein VIII, (minor capsid protein) [35], and E1A (an early antigen). We selected the top-scoring 2% of sequences for synthesis, and included published epitopes, resulting in 29 peptides for the HLA-A*02, 21 for HLA-A*01 and 21 for HLA-A*24 (Table S1 in File S1). All peptides were analyzed for their capacity to stimulate CD8^+^ T-cells as defined by IFN-γ- ELIspot detection. To avoid overlooking HAdV-specific T-cells with very low frequency, PBMCs from at least 16 appropriate donors sharing the HLA allele presenting the peptide were stimulated with the peptide together with IL-2, and expanded for 13 days (Fig. 1A). Peptides were defined as immunodominant if the ELIspot response was specifically positive in more than 50% of appropriate, and negative in most inappropriate donors. These criteria were fulfilled for the novel E1A-based peptide LLD (A*02) as well as for the known hexon-peptides LTD (A*01), and TYF (A*24) which showed an IFN-γ response in 49/74, 68/73, and 44/58 cases, respectively (Fig. 1B, and Table S1 in File S1). All other known hexon-based peptides for YVL and TFY (both A*02) mediated either low responses or nonspecific recognition (Fig. 1C).

### Applicability of multimers to detect very rare HAdV-specific T-cells in healthy donors

Using the LLD-based HLA-A*02 streptamer we assessed the functionality of this novel and of four other known epitopes complexed in multimers of HLA-types A*01, A*24, B*07 and B*35. Due to the very low frequency of HAdV-specific T-cells in freshly drawn blood, we cultured PBMCs for 6 to 12 days using HAdV-specific 9-mer peptides or 15-mer pepmixes and IL-15 as stimulants (Fig. 1D). After 12 days of expansion, A*02 streptamer-positive T-cells were reliably detectable in 5/5 HLA-A*02 positive donors, with frequencies ranging from 0.6 to 8.6% of CD8^+^ T cells, representing an increase of 0.8 to 2 logs as compared to day 0 (Fig. 1E). The specificity of the new A*02-streptamer was confirmed by its failure to bind to CD4^+^ T-cells in HLA-A*02 matched, and to CD8^+^ T-cells in HLA-A*02 mismatched donors (data not shown). In addition, specific killing between 38% and 74% of A*02-multimer purified-T-cells could be achieved, if LLD peptide-loaded target cells were used with different peptide concentrations. No specific killing was observed when A*02-mismatched target cells were used (Figure 1F). Regarding the known epitopes, only 5/25 donors were determined positive with multimers on day 0 (range 0.06–0.2% of CD8^+^ T-cells). On days 6 and 12, 12/13 and 25/25 of the matched donors were clearly positive, with a range from 0.09–2.1% to 0.64–28% of CD8^+^ T cells (Fig. 1G), representing a 1–3 log increase in the frequency of multimer-positive T-cells.

To address cross reactivity, we tested whether common subgroup-C-derived HAdV-streptamers detect HAdV-specific T-cells specific for subgroups A–F. Therefore, PBMCs were expanded for 12 days with peptides derived from hexon proteins of different HAdV subgroups (A–F), followed by staining with subgroup-C-derived HAdV-streptamers. The subgroup-C-derived streptamers restricted to A*01 and B*07 did detect HAdV-specific T-cells from all six different subgroups. For the A*24- and B*35-subgroup-C-derived streptamers, HAdV-specific T-cells were neither detectable from subgroups A and B nor from A and F (Fig. 1H). Notably, the new E1A-derived A*02 peptide is not conserved in other subgroups, as determined by the basic local alignment search (blast), and can therefore not be employed to detect HAdV-specific T-cells from other subgroups (data not shown).

Beside HAdV, similar results with streptamers were seen for the detection of EBV and BKV-specific T-cells, before and after expansion (Figure A and B in Figure S1 in File S1). Whereas all 6 EBV-streptamers showed reliable staining results (Figure. A in Figure S1 in File S1) for BKV, only the B07-restricted streptamer (Figure B in Figure S1 in File S1) was functional. These data indicate that novel and known multimers, in combination with a short *in vitro* expansion period, is a reliable tool to monitor virus-specific T-cells.

In analogy to the multimer results, a short expansion period was also necessary to detect HAdV-, EBV- and BKV-specific T-cells when using the IFN-γ-CSA (Figure C – E in Figure S1 in File S1).

### Reconstitution of HAdV-specific T-cells in the context of HAdV infection following HSCT

**Figure 2:**
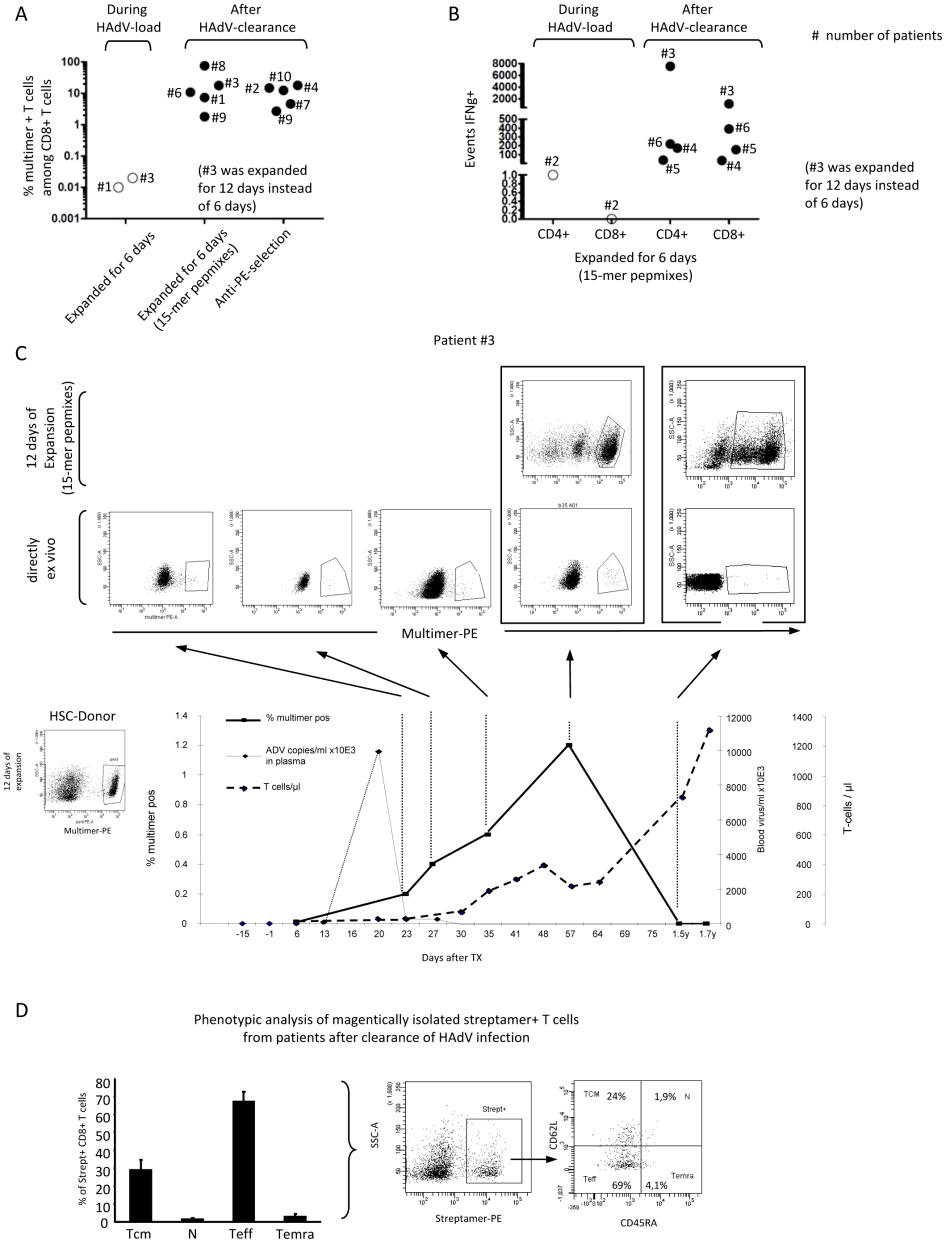
Analysis of HAdV-specific T-cells in patients during and after allogeneic SCT. The presence of 6 day expanded or magnetically isolated HAdV-specific T-cells was assessed by A) the percentage of multimer^+^ T-cells among CD8^+^ and by B) events of IFN-γ secreting CD4^+^ and CD8^+^ T-cells during HAdV load, or after ADV clearance in 10 patients. Of note, for most patients, either multimer or IFN-γ assays before or after viral load were performed. Each dot refers to the appropriate patient number, as indicated.C) Detailed analysis of ADV-multimer^+^ T-cells of patient No. 3 and the representative HSC-donor are shown. Dot plots show the percentage of HAdV-multimer^+^ T-cells among CD8^+^ at several time points after clearance of HAdV plasma load. For the last two stainings, cells were measured directly *ex vivo* and after a 12 instead of 6 day expansion period. The graphs show percentage of HAdV-multimer^+^ T-cells among CD8^+^ (bold line, rectangle), HAdV copies per ml serum (dotted line, diamond) and number of CD3^+^ T-cells/µl blood (bold line, diamond). D) A summarizing diagram + SEM (of patients No. 2, 4, 7, 9 and 10) including representative dot plots of magnetically isolated HAdV-streptamer^+^ T-cells and percentages of their 4 subsets of naïve (N), central (TCM), effector memory (TEM) and effector memory CD45RA^+^ (TEMRA) T-cells are shown.

By applying the expansion protocol for diagnosis, we assessed whether the presence of CD8^+^ and/or CD4^+^ HAdV-specific T-cells in patients correlated with clearance of adenoviral load in stool and blood. All blood samples were from 10 patients positive for HAdV in stool (range 6×10^2^–2×10^10^copies/gram), 3 of them were also viremic (range 7×10^2^–6×10^7^ copies/ml). T-cell analyses performed in 2 patients with multimers and in 1 patient with the IFN-↖-CSA during HAdV-infection showed no detectable HAdV-specific T-cells (Fig. 2A and B). After clearance, however, samples from 9/9 patients stained with multimers (after expansion or magnetic selection) showed positive results. In 4/4 cases, CD8^+^ and CD4^+^ HAdV-specific T-cells were also detectable by the IFN-γ-CSA (Fig. 2A and B and Table 1). Of note, HAdV-specific T cells had been detectable in all respective donors prior to stem cell donation (data not shown). More detailed analysis of patient 3 demonstrated that, shortly after viral clearance, multimer^+^ HAdV-specific CD8^+^ T-cells were clearly detectable at day 23 post HSCT and further increased during the following days (Fig. 2C). After 18 months, however, a clear population of HAdV-specific T-cells was only seen after short term cell expansion (Fig. 2C).

**Table 1:** Patient characteristics.

Patient no.	Sex	Age at TX	Diagnosis	Donor	Source	Conditioning	GVHD prophylaxis	PCR-positive results in stool, first and last day	highest PCR-positive results in stool, copies/g	PCR-positive results in blood, first and last day	HAdV strain	Antiviral treatment	CD3+ >50/µl post TX, day	Detection of ADV-specific T cells during ADV-clearance, yes/no	Detection of ADV-specific T cells after ADV-clearance, yes/no	Status at month 6 post TX concerning ADV infection
1	w	19	T-ALL	FD, haplo, m T cell depletion	PBSC	Flu/VP16/OKT3	mmf	day 102–272	5×10E7	day 109–110, 6×10E5	C	Cidofovir	between day 34–41	no	yes	died after Heart TX, no ADV infection
2	m	2	M Kostmann	MUD, m	BM	Flu/Thio/Mel/ATG	CyA, mmf	day 21–260	2×10E7	not positive	C	Cidofovir	between day 14–21	no	yes	still alive, no ADV infection
3	w	3	MHC II Deficiency	MFD, f	BM	Flu/Thio/Mel/ATG	CyA, mmf	day –15–75	1×10E10	day 13–27, 1×10E4	C	Cidofovir + Ribavirin	between day 13–20	no	yes	still alive, no ADV infection
4	w	8	NBL Rez.	FD, haplo, m T cell depletion	PBSC	Flu/Thio/Mel/OKT3	CyA	day 106–252	1×10E6	not positive	C	none	between day 27–41	not determined	yes	still alive, no ADV infection
5	m	12	ALL Rez.	MUD, m	BM + Boost (day154)	TBI/VP16/ATG	CyA, MTX	day 21–181	2×10E10	day 27–49, 1×10E3	A and C	Cidofovir	between day 28–34	not determined	yes	still alive, no ADV infection
6	m	5	Sept Granulomatose	MFD, f	BM	Flu/Thio/Mel/ATG	CyA, mmf	day –11–92	2×10E5	not positive	C	none	between day 13–20	not determined	yes	still alive, no ADV infection
7	m	7	NBL IV	FD, haplo, f T cell depletion	PBSC	Flu/Thio/Mel/OKT3	CyA	day 6–118	5×10E7	not positive	B and C	Cidofovir	between day 34–42	not determined	yes	still alive, no ADV infection
8	w	3	Hyper IGE Syndrom	MUD, f	BM	Flu/Thio/Mel/ATG	CyA, mmf	day 5–13	1×10E4	not positive	C	Gancyclovir	between day 13–16	not determined	yes	still alive, no ADV infection
9	m	5	Fanconi, MDS	MFD, f	BM	FLU/BU/ATG/Campath	CyA, mmf	day 11–281	9×10E6	not positive	A and C	Ribavirin	between day 14–18	not determined	yes	still alive, no ADV infection
10	m	6	C ALL Rez.	MUD, f	BM	TBI/VP16/ATG		day 54–61	7×10E3	not positive	C	none	between day 19–22	not determined	yes	still alive, no ADV infection

Patient No. 2 had transient enteritic symptoms attributed to clostridium difficile infection;

Patient No. 10 had transient enteritic symptoms attributed to gut GVHD.

GVHD, graft-versus-host disease; ALL, acute lymphoblastic leukemia; Rel, relapse; Morbus Kostmann, MHC II, major histocompatibility complex class II, NBL rel, Neuroblastoma relapse; Sept, septic granulomatous disease; NBL IV, Neuroblastoma grade IV; IGE, immunoglobulin E; MDS, myelodysplastic syndrome; common ALL, FD, family donor; haplo, haploidentical; MUD, matched unrelated donor; MFD, matched family donor; m, male; f, female; PBSC, peripheral blood stem cells; BM, bone marrow; Flu, fludarabine; VP16, Etoposide; ThioMel, Thiotepa Melphalan ATG, antithymocyte globulin; TBI, total body irradiation; mmf, mycophenolate mofetil; CyA, cyclophosphamide A; PCR, polymerase chain reaction; HAdV, adenovirus.

To determine the phenotype of CD8^+^ HAdV-specific T-cells in patients who had cleared adenoviral infection several months before, multimer^+^ HAdV-specific T-cells were magnetically enriched prior to analysis. HAdV-multimer^+^ T-cells revealed clear populations of central memory T-cells (TCM) (median: 30%) and effector memory T-cells (TEMs) (median: 65%) emphasizing a prominent role of TCMs in maintaining long-term immunity in patients (Fig. 2D). Taken together, these results show that CD4^+^ and CD8^+^ HAdV-specific T-cells include high proportions of TCMs, and that their presence correlates with clearance of HAdV load in patients.

### Generation and phenotypic characterization of seHAdV-T-cells for potential clinical use

**Figure 3:**
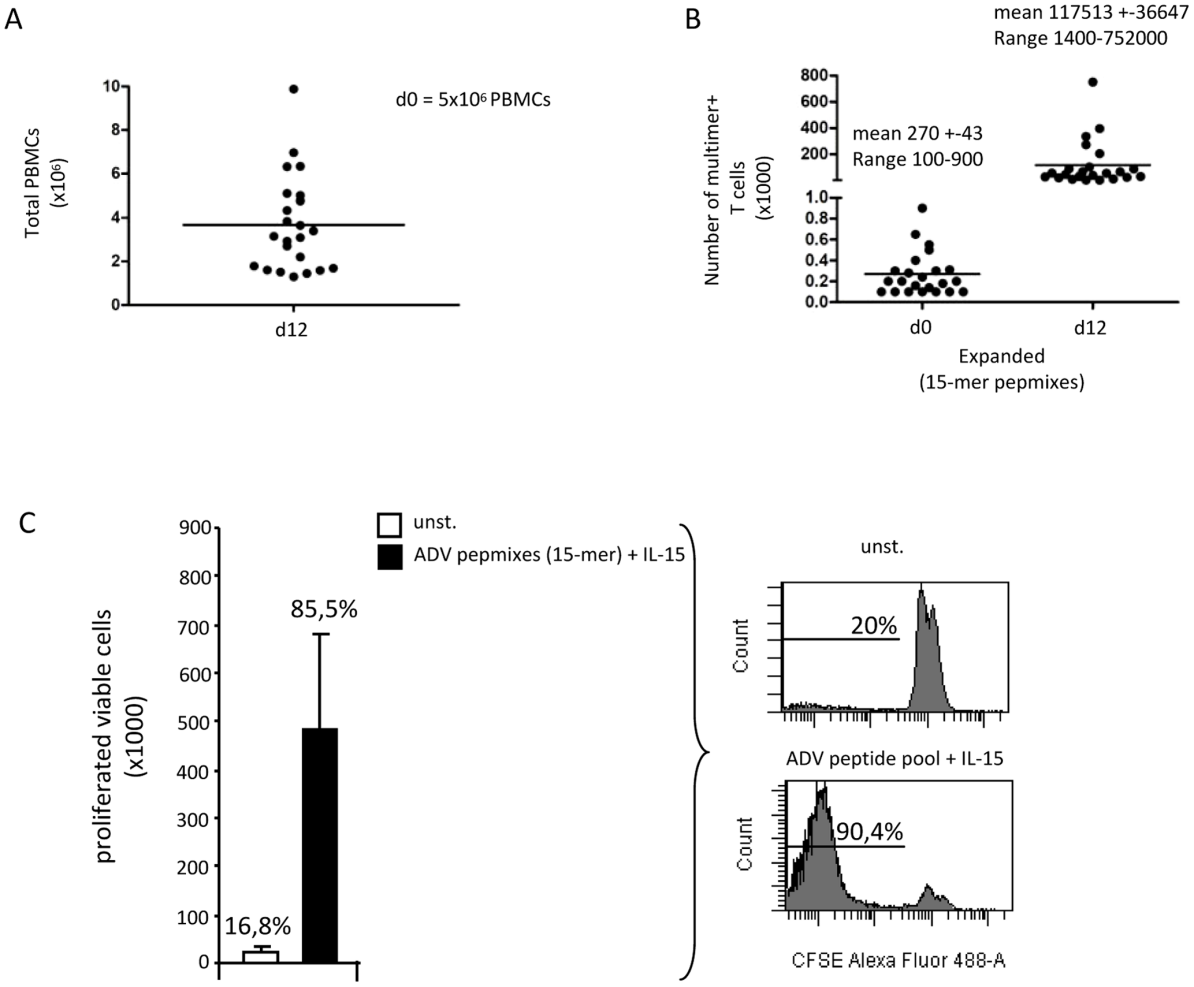
Generation and detailed phenotypic analysis of seHAdV-T-cells. PBMCs (5×10^6^/12 well) were expanded for 12 days using the HAdV peptide pool and IL-15, as described in material and methods. A) The total number of PBMCs after expansion, and B) the absolute number of HAdV-multimer-specific T-cells at days 0 and 12, are shown from 24 donors, including mean +SEM and ranges. C) PBMCs (2.5×10^6^/24 well) were CFSE-labeled without stimulation (unst.), or stimulated with the HAdV-peptide pool in the presence of IL-15, as described in Materials and Methods. A summarizing diagram + SEM of 3 donors shows cell number (x1000) (bars) and percentage (above bars) of viable proliferating cells after expansion including a representative histogram.

Based on the protocol used for diagnosis (Fig. 1D), we generated seHAdV-T-cells (short-term expanded human adenovirus-specific T cells) with GMP-compliant adaptable peptide mixes and analyzed the absolute cell number and function in more detail. Whereas the starting cell number of 5×10^6^ PBMCs was slightly reduced to a median of 3.7×10^6^ cells after 12 days of expansion (Fig. 3A), the total number of HAdV-streptamer^+^ T-cells increased 435 fold from 270 to 117513 (Fig. 3B). For clinical use, the number of starting fresh/frozen PBMCs can be easily scaled up to 25×10^6^ PBMCs (instead of 5×10^6^),which should result in sufficiently high cell numbers for both treatment and quality control analyses.

To assess the percentage values and total cell numbers of viable proliferating seHAdV-T-cells after *in vitro* expansion in 24 well plates, PBMCs were labeled with CFSE prior to culture. Compared to unstimulated controls (total cell number (×1000): 21±13.8 or percentage value: 16.8), HAdV-peptide pool and IL-15-driven stimulation resulted in high numbers (×1000) (479,5±194) and percentage values (85.5%) of proliferating viable T-cells (Fig. 3C). But also the number of HAdV-streptamer^+^ T-cells/µl within the culture was high (mean: 33.2/µl) in HAdV-peptide pool- and IL-15 stimulated cells, whereas HAdV-specific cells were not detectable in unstimulated or control peptide(MAGE-A1)-stimulated PBMCs (data not shown).

**Figure 4:**
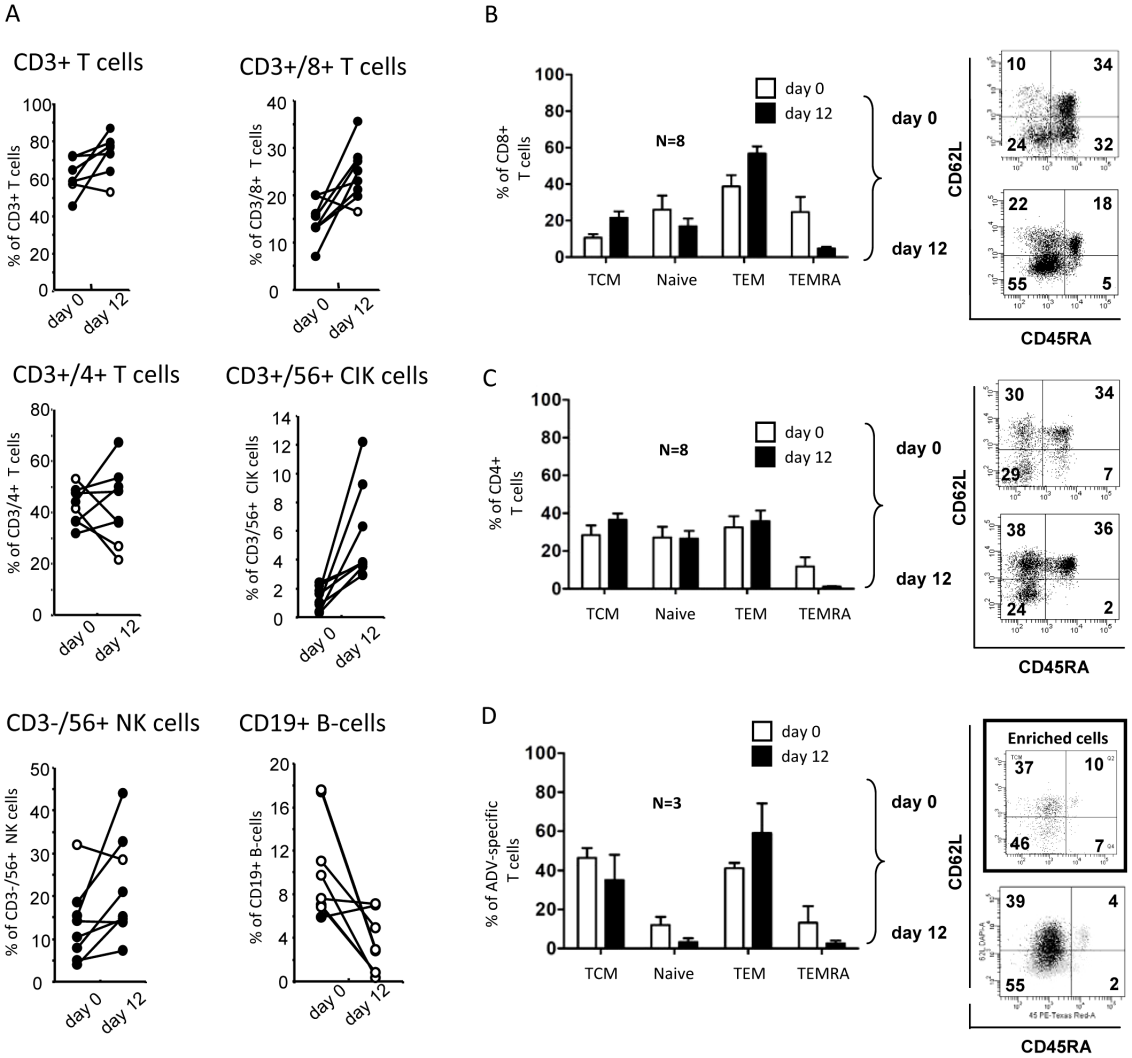
Phenotypic analysis of seHAdV-T-cells. A) Percentage values of different cell populations (as indicated, including cytokine-induced killer cells (CIK)) within PBMCs, before (day 0) and after expansion (day12) of 8 donors. Percentage values of TCM (upper left), Naïve (upper right), TEM (lower left) and TEMRA (lower right) T cell populations within CD8^+^ (B), CD4^+^ C), and HAdV-streptamer^+^ T-cells D) on days 0 (white bars) and 12 (black bars). The graph shows mean+SEM of 8 (for CD4^+^ and CD8^+^ T-cells) and 3 (for HAdV-streptamer^+^ T-cells) donors. Notably, for phenotypic analysis of HAdV-streptamer^+^ T cells on day 0 (white bars), beads-based magnetic isolation was performed. Representative dot plots are shown.

Phenotypic analysis revealed that, after expansion, 71%±3.8 were CD3^+^ T-cells including 24%±2 CD8^+^ T-cells and 42.4%±5.3 CD4^+^ T-cells (Fig. 4A). Whereas the percentage of TCMs (21.6%±3.5) and TEMs (56.8%±3.9) was slightly increased in CD8^+^ T-cells, no significant change was seen within CD4^+^ T-cells (Fig. 4B and C). The percentage of TCMs within the streptamer^+^ population was also not significantly altered (Fig. 4D), indicating that the expansion procedure did not lead to terminal cell differentiation. Of note, cultures supplemented with IL-15 showed highest numbers of HAdV-multimer^+^ T-cells/µl (3.5-fold increase) compared to cultures stimulated in the absence of cytokines, and a 2-fold increase compared to IL-2 or IL-7 stimulation, but no detectable influence on their phenotype (data not shown).

### Strongly reduced proliferative capacity of seHAdV-T-cells upon alloantigen stimulation

**Figure 5:**
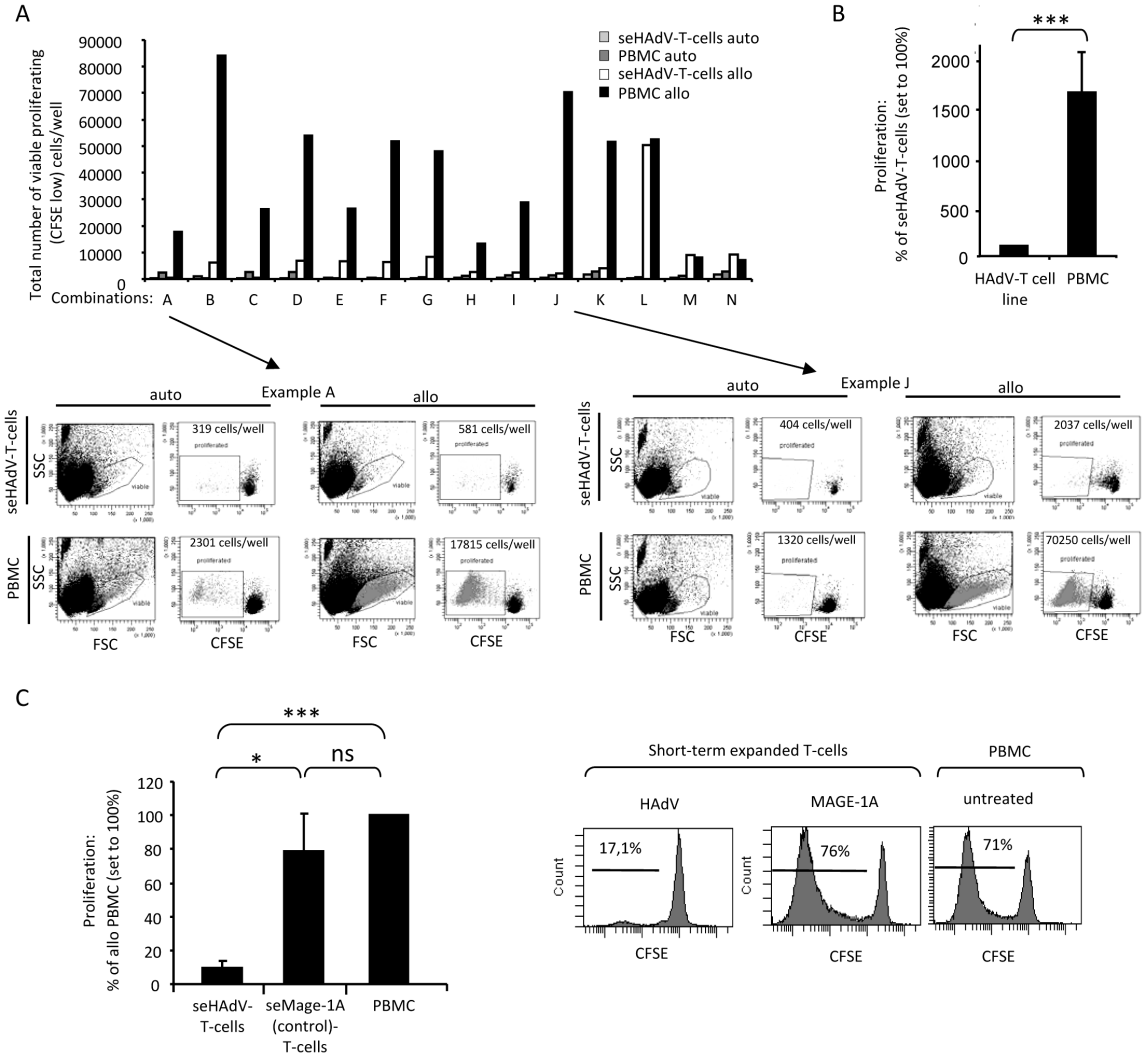
Alloreactive potential of PBMCs versus expanded control- and seHAdV-specific T-cells. CFSE-labeled autologous or allogeneic responder cells (PBMCs or seHAdV-T-cells) were mixed 1∶1 with irradiated stimulator cells (PBMCs) and incubated for 7 to 8 days. A) The graph shows the total number of proliferated viable cells per 96 well plate of 14 different donor/recipient combinations A–N). Proliferation of seHAdV-T-cells or PBMCs in response to autologous (auto) or allogeneic (allo) irradiated PBMCs are shown. In addition, representative examples from donors A and J are given. B) A summarizing graph show mean + SEM from all combinations (except for L,M and N); The percentage of viable proliferating seHAdV-T-cells was set to 100% and calculated based on total cell number. C) seHAdV-T-cells or seMAGE-1A-T-cells (control) and PBMC were mixed with allogeneic irradiated stimulator PBMCs. A summarizing graph shows mean + SEM of 4 combinations. First, total cell number of viable proliferating (CFSE-low) seHAdV-, seMAGE-1A-T-cells and PBMCs/well are calculated. Based on these total cell number, the percentage values were analyzed and compared to allogeneic PBMCs, which was set to 100%. A representative histogram of one donor is shown, including percentage values of proliferated cells. Significance vs HAdV-T-cell line: o, p< = 0.07; *, p< = 0.01.***, p< = 0.001.

Next we tested the alloreactive potential of seHAdV-T-cells. In 11 out of 14 allogeneic-pairs, the mean proliferative response of allogeneic PBMCs was about 1.2 log higher compared to that of seHAdV-T-cells (Fig. 5A and B). Only 3 combinations showed comparable residual alloreactivity to allogeneic-PBMCs (combination: L, M, N) (Fig. 5A). Of note, a closer look at HAdV-multimer^+^ T-cells within the culture revealed that they divided only once representing low if any alloreactivity (data not shown). In addition, we could show that the median alloreactive potential of control short-term expanded T-cells (stimulated with MAGE-1A and IL-15) was similar to that of PBMCs, and 0.9 log higher than that of seHAdV-T-cells (Fig. 5C). These results support our observation that the alloreactive potential of seHAdV-T-cells is strongly reduced after *in vitro* expansion.

### seHAdV-T-cells are highly functional and specific, and fail to kill unpulsed-allogeneic target cells

**Figure 6:**
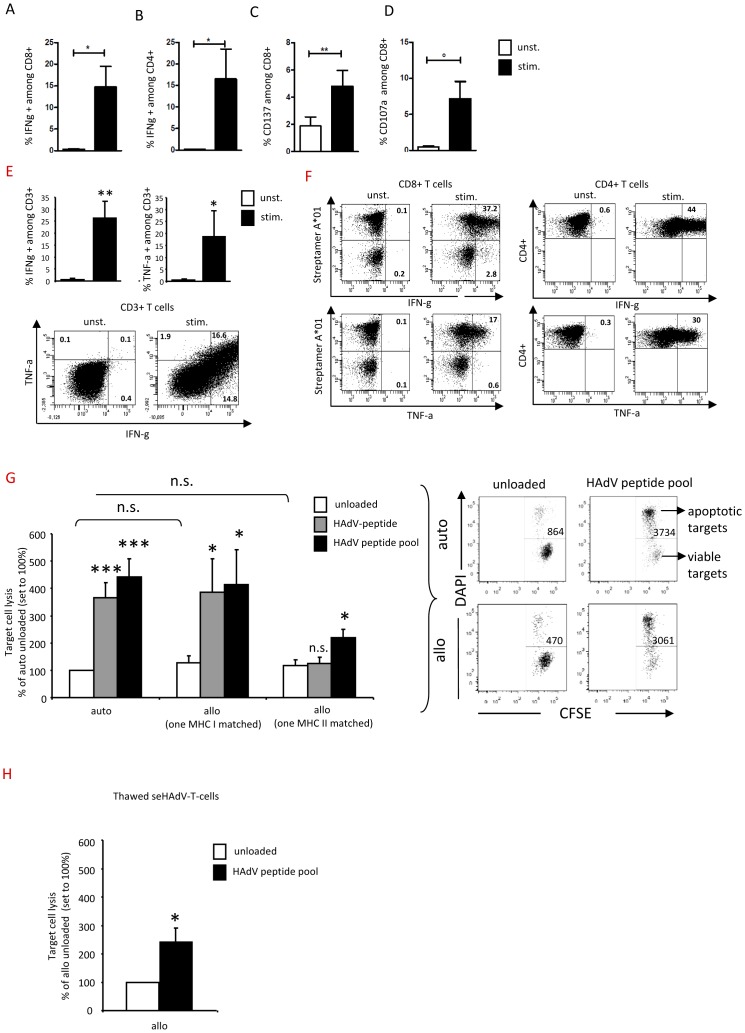
Functional and cytolytic activity of seHAdV-T-cells. seHAdV-T-cells were used for the cytotoxic assay. The percentage values of IFN-γ, CD137 and CD107a expressing seHAdV-T-cells among CD4^+^ and/or CD8^+^ are shown A–D) as indicated. E) The percentage values of IFN-γ and TNF-α among CD3+ cells are shown, including representative dot plots indicating simultaneous expression. F) Representative dot plots of IFN-γ and TNF-α-expressing CD8^+^ T-cells including streptamer^+^ T cells and CD4^+^ T-cells. G) Specific lysis is shown of autologous and allogeneic PHA-blasts (target cells) matched in only a single MHC I, or in only a single MHC II, unloaded (white bar), HAdV-peptide- (grey bar) or HAdV-peptide pool (black bar)-loaded, induced by seHAdV-T-cells. The total number of “dying” target cells/well was evaluated from each sample. Based on these, a summarizing graph shows the percentage of dying target cells related to unloaded autologous (auto) target cells which is set to 100%. Representative dot plots, indicating “dying” cells/well, are shown. H) Specific lysis of allogeneic, unloaded (white bar) or HAdV-peptide pool (black bar)-loaded target cells induced by post-thaw seHAdV-T-cells. Based on total cell number of dying cells, a summarizing graph shows the percentage of dying target cells related to unloaded target cells, which is set to 100%. Significance vs unloaded autologous targets: ns = not significant, *, p< = 0.01.***, p< = 0.001.

The capacity of seHAdV-T-cells to express activation and cytotoxic markers, such as IFN-γ, CD137 and CD107a, was highly increased after restimulation with HAdV-pepmix-pulsed monocytes (Fig. 6A–D). Furthermore, seHAdV-T-cells produced both IFN-γ and TNF-α (Fig. 6E and F). The lysis of peptide or of peptide-pool-pulsed autologous target cells by seHAdV-T-cells was about 4-fold higher as compared to unpulsed targets (Fig. 6G). Similar lysis was seen when HAdV-pulsed-allogeneic target cells, matched in only one MHC class I or II antigen, were used (Fig. 6G). Unpulsed or control (CMV)-pulsed autologous or allogeneic targets were not recognized, which strongly supports their specificity and the loss of alloreactivity of seHAdV-T-cells (Fig. 6G and data not shown). This cytotoxic activity was also maintained in post-thaw seHAdV-T-cells (Fig. 6H).

### Generation of short-term-expanded virus-specific T-cells against several viruses for potential clinical use

Our protocol to generate seHAdV-T-cells was adapted to expand also EBV-, CMV- and BKV-specific T-cells. Whereas the median absolute number of cells was not significantly altered after expansion, the total cell number of streptamer^+^ T-cells was 1–2 log increased, irrespective of the type of virus-specific T-cells (Figure A, D, and G in Figure S2 in File S1). As seen for HAdV, the proportions of TCM and TEM of CD4^+^ and CD8^+^ virus-specific T-cell lines hardly changed during the expansion period (Figure B, E, and H in Figure S2 in File S1). Specific lysis by all cell lines was only observed for peptide-loaded autologous and allogeneic, but not for unloaded allogeneic target cells mismatched or matched in only 3 alleles. (Figure C, F, and J in Figure S2 in File S1).

## Discussion

PCR screening for HAdV following allogeneic HSCT allows early detection of impending invasive HAdV-infections, and timely preemptive antiviral treatment [8], [36]. Recently, it has been shown that the reconstitution of HAdV-specific T-cells plays a pivotal role in the clearance of HAdV infection [11], [12], [13], [14]. Few groups suggested that combined monitoring of viral load and virus-specific immunity by ELIspot, tetramer staining or the IFN-γ-CSAs has a clinical impact on the therapeutic intervention for pediatric allogeneic HSCT patients [36], [37], [38], [39]. One of the most sensitive and fastest tools to monitor virus-specific T cells are multimers. Therefore, efforts have been made over the past years to identify HAdV-derived MHC class I-restriced as well as MHC class II-restricted epitopes [14], [27], [40], [41]. Nevertheless, the number of published class I epitopes remained rather low, and the focus has been on hexon, the main capsid protein of the virion. Our analyses of different proteins identified an immunodominant A*02-restricted HAdV subgroup C-specific epitope derived from the E1A protein. Based on this epitope, a functional multimer was produced that showed reliable staining results. To our knowledge, this is the first functional A*02-restricted multimer specific for HAdV. Other promising previously published A*02-restricted candidates failed to be useful for the production of functional multimers. Although in a pilot study the treatment of a single patient with pentamer+ CD8+ T-cells specific for HAdV was not successful [22], more studies will be necessary to determine whether polyclonal CD4^+^ and CD8^+^ HAdV-specific T-cells are necessary for successful immunotherapy.

However the detection of HAdV-specific T-cells is hampered by the low frequency of HAdV-specific T-cells in peripheral blood [14], [17], [18]. Even with the more sensitive ELIspot technique (procedure needing 3 days), false negative results cannot be excluded if uncultured PBMCs are used [29]. The prevalence of HAdV in the Caucasian population is supposed to be above 80% [32]. We demonstrated that in most cases a 6 day expansion period was sufficient to obtain clearly positive multimer-based results in 90% of donors. Similar results were seen when the IFN-γ-CSA instead of streptamers was used after expansion. Moreover, in addition to the generally known cross-reactivity of reactive T-cells between HAdV-subgroups [24], we could show that A*24 and B*35 streptamers are not able to recognize subgroup A- or subgroup A and F-derived HAdV-specific T-cells, respectively. This is a very important piece of information, if HAdV-multimers are supposed to be used for diagnosis or future multimer-based therapies.

So far, only one study including seven pediatric and six adult patients showed that, besides CD4^+^ [7], [12], [13], also CD8^+^ virus-specific T-cells [14] are detectable after HAdV clearance in patients after HSCT. We confirmed these results with additional 10 pediatric patients, using the IFN-γ-CSA or multimer analysis of expanded or magnetically selected cells. Out of 7 patients who were HAdV positive in stool and HAdV-negative in plasma, two had transient enteritic symptoms, which were attributed to *Clostridium difficile* infection and gut GvHD respectively, and five remained asymptomatic. HAdV-specific T-cells were detectable in all patients after clearance of infection. Of note, 3/10 patients who cleared HAdV infection, had received T-cell depleted grafts from haploidentical donors (<10^5^ T-cells/kg BW). This may indicate that, even in the context of profound lymphopenia, viral antigen can trigger virus-specific immune responses. None of these patients reactivated HAdV infection within several months after HSCT, which could be due to the presence of residual HAdV-specific T-cells with characteristics of TCM and TEMs, as seen in all patients analyzed. It further strengthens the suggested important role of TCMs in mediating long-term protection [16], [42]. Although the optimal time point for a T-cell immunotherapy in clinical practice is unknown, recent data suggest that the generation or isolation of virus-specific T-cells should occur within the first weeks after detection of a high viral load (>10^6^ copies) in stool in order to allow prompt treatment in case of invasive HAdV infection [8], [18], [43], [44].

Addressing these criteria, we used 5×10^5^ fresh or frozen PBMCs (including 2.5 to 3.5×10^6^ CD3+T cells) as starting material and managed to increase the number of HAdV-streptamer^+^ T-cells 435-fold within 12 days. The slightly reduced cell number from 5 to 3.7×10^6^ could be explained by the delayed stimulation with IL-15 and loss of unspecific T cells during the culture period. Of note, prophylactic cryo-preservation of PBMCs from appropriate donors prior to HSCT proved helpful to safe time. Culture of both fresh and frozen PBMCs resulted in high values of total proliferating cells (up to 85.5%) and at least 68.8±8%, if only highly proliferating cells were gated (data not shown). By using a similar but more strict gating strategy, an approach by Gerdemann et al., who also used peptide mixes but preferentially combined with IL-4 and IL-7, resulted in 52.4% of proliferating cells. In contrast to this work, natural killer (NK) and cytokine-induced killer (CIK) cells, which are assumed to be beneficial in the prevention and treatment of relapses[45], [46], are still present after 12 days of expansion. Although our work shares certain features with the protocol by Gerdemann et al., it differs considerably with regard to the stimulation procedure itself, and consequently also with regard to the nature of expanded T-cells and focuses mainly on the safety of our seHAdV-T-cells, as mentioned above.

In our experiments, all seHAdV-T-cells were highly functional, and not only able to lyse antigen-pulsed autologous but also antigen-pulsed allogeneic target cells, if at least one MHC class I or II was matched, which, to our knowledge, had not been shown in that detail before and could be of high relevance for haploidentical or third party donors. For IL-4- and IL-7-driven expansion of HAdV-specific T-cells, at least a 16 day expansion period was necessary to obtain sufficient cytolytic activity [31]. In contrast to previous studies, the killing of MHC class I-matched HAdV-pulsed targets was much higher compared to MHC II-matched targets, indicating that mostly HAdV-specific CD8^+^ T-cells are involved. This discrepancy might be explained by the use of 15mer instead of 30mer peptides, since the 30mer peptides were described to contain predominantly CD4^+^ epitopes [29], [47]. In addition, even post-thaw seHAdV-T-cells were able to kill partially matched allogeneic target cells albeit to a lower extend compared to fresh seHAdV-T-cells. This finding would enable the prophylactic generation and infusion on demand.

To address the potential risk of seHAdV-T-cells to induce GvHD, the percentage of residual unspecific T-cells - represented by non-proliferating T-cells during the culture period - was analyzed. Although about 15% of the seHAdV-T-cell population did not proliferate as indicated by their unchanged high CFSE load, MLRs showed that, in at least 11/14 donor/recipient pairs, the alloreactive potential of seHAdV-T-cells was reduced by 1.2 log compared to unmanipulated PBMCs. Only 1/14 cases (combination L) showed alloreactivity signals similar to those of the control PBMCs, although in the cytotoxic assay no significant alloreactivity was seen (data not shown). For the other two, the MLR might have failed since the alloreactivity of PBMCs was near the background level. However, residual alloreacivity in some combinations could also be explained by the fact that, in contrast to other studies [16], [29] only one MHC allele was matched. Reduced or even absent alloreactivity of seHAdV-T-cells was further confirmed by comparison with control T-cells expanded with MAGE-A1-peptide pools, or by the failure to recognize and lyse allogeneic target cells. The fact that residual alloreactivity, despite expansion, can never be completely excluded, was also shown by other groups [16], [18], [29]. In addition, Chen et al. showed that the capacity of *in vitro* expanded alloreactive T-cells to survive and expand *in vivo* is limited [48]. This was also supported by Melenhorst et al. who showed no correlation between *in vitro* results and *in vivo* data concerning alloreactivity [49]. Clinical evidence supports that even a small number of virus-specific T-cells (like 10^3^ to 10^4^/kg body weight), which is easily achievable with our protocol, is sufficient to attain therapeutic efficacy, and infusion of such low lymphocyte numbers would further minimize the risk for GvHD [18]. By adapting our short-term expansion protocol, we were also able to generate high numbers of functional virus-specific T-cells directed against CMV, EBV, and BKV. Also these cells were able to lyse autologous and allogeneic peptide-loaded target cells, although, in some cases, significant lysis was hindered by the high intra-individual variability.

In conclusion, we optimized tools for diagnosis of HAdV-specific T-cells and underline the importance of CD8^+^ HAdV-specific T-cells in the clearance of HAdV-load in patients. In addition, we were able to generate efficient virus-specific T-cells mainly against HAdV but also CMV, EBV, and BKV within 12 days. The usage of fresh or frozen PBMCs further enables an immunotherapy protocol within a short time span, with low cost and effort, and with the potential for broad clinical application.

## Supporting Information

File S1 **Figure S1, Figure S2, Table S1 and Table S2.**
(DOC)

## References

[1] Lion T, Baumgartinger R, Watzinger F, Matthes-Martin S, Suda M, et al (2003) Molecular monitoring of adenovirus in peripheral blood after allogeneic bone marrow transplantation permits early diagnosis of disseminated disease. Blood 102: 1114–1120. 12702513 10.1182/blood-2002-07-2152

[2] Gooley TA, Chien JW, Pergam SA, Hingorani S, Sorror ML, et al (2010) Reduced mortality after allogeneic hematopoietic-cell transplantation. N Engl J Med 363: 2091–2101. 21105791 10.1056/NEJMoa1004383 PMC3017343

[3] Watcharananan SP, Kiertiburanakul S, Piyatuctsanawong W, Anurathapan U, Sungkanuparph S, et al (2010) Cytomegalovirus, adenovirus, and polyomavirus co-infection among pediatric recipients of allogeneic stem cell transplantation: characteristics and outcome. Pediatr Transplant 14: 675–681. 20412509 10.1111/j.1399-3046.2010.01325.x

[4] George D, El-Mallawany NK, Jin Z, Geyer M, Della-Latta P, et al (2012) Adenovirus infection in paediatric allogeneic stem cell transplantation recipients is a major independent factor for significantly increasing the risk of treatment related mortality. Br J Haematol 156: 99–108. 22008222 10.1111/j.1365-2141.2010.08468.x

[5] Schilham MW, Claas EC, van Zaane W, Heemskerk B, Vossen JM, et al (2002) High levels of adenovirus DNA in serum correlate with fatal outcome of adenovirus infection in children after allogeneic stem-cell transplantation. Clin Infect Dis 35: 526–532. 12173125 10.1086/341770

[6] Symeonidis N, Jakubowski A, Pierre-Louis S, Jaffe D, Pamer E, et al (2007) Invasive adenoviral infections in T-cell-depleted allogeneic hematopoietic stem cell transplantation: high mortality in the era of cidofovir. Transpl Infect Dis 9: 108–113. 17461995 10.1111/j.1399-3062.2006.00184.x

[7] Myers GD, Krance RA, Weiss H, Kuehnle I, Demmler G, et al (2005) Adenovirus infection rates in pediatric recipients of alternate donor allogeneic bone marrow transplants receiving either antithymocyte globulin (ATG) or alemtuzumab (Campath). Bone Marrow Transplant 36: 1001–1008. 16184180 10.1038/sj.bmt.1705164

[8] Lion T, Kosulin K, Landlinger C, Rauch M, Preuner S, et al (2010) Monitoring of adenovirus load in stool by real-time PCR permits early detection of impending invasive infection in patients after allogeneic stem cell transplantation. Leukemia 24: 706–714. 20147979 10.1038/leu.2010.4

[9] Hoffman JA, Shah AJ, Ross LA, Kapoor N (2001) Adenoviral infections and a prospective trial of cidofovir in pediatric hematopoietic stem cell transplantation. Biol Blood Marrow Transplant 7: 388–394. 11529489 10.1053/bbmt.2001.v7.pm11529489

[10] Lankester AC, Heemskerk B, Claas EC, Schilham MW, Beersma MF, et al (2004) Effect of ribavirin on the plasma viral DNA load in patients with disseminating adenovirus infection. Clin Infect Dis 38: 1521–1525. 15156436 10.1086/420817

[11] Chakrabarti S, Mautner V, Osman H, Collingham KE, Fegan CD, et al (2002) Adenovirus infections following allogeneic stem cell transplantation: incidence and outcome in relation to graft manipulation, immunosuppression, and immune recovery. Blood 100: 1619–1627. 12176880 10.1182/blood-2002-02-0377

[12] Feuchtinger T, Lucke J, Hamprecht K, Richard C, Handgretinger R, et al (2005) Detection of adenovirus-specific T cells in children with adenovirus infection after allogeneic stem cell transplantation. Br J Haematol 128: 503–509. 15686459 10.1111/j.1365-2141.2004.05331.x

[13] Heemskerk B, Lankester AC, van Vreeswijk T, Beersma MF, Claas EC, et al (2005) Immune reconstitution and clearance of human adenovirus viremia in pediatric stem-cell recipients. J Infect Dis 191: 520–530. 15655775 10.1086/427513

[14] Zandvliet ML, Falkenburg JH, van Liempt E, Veltrop-Duits LA, Lankester AC, et al (2010) Combined CD8+ and CD4+ adenovirus hexon-specific T cells associated with viral clearance after stem cell transplantation as treatment for adenovirus infection. Haematologica 95: 1943–1951. 20562315 10.3324/haematol.2010.022947 PMC2966918

[15] Einsele H, Roosnek E, Rufer N, Sinzger C, Riegler S, et al (2002) Infusion of cytomegalovirus (CMV)-specific T cells for the treatment of CMV infection not responding to antiviral chemotherapy. Blood 99: 3916–3922. 12010789 10.1182/blood.v99.11.3916

[16] Leen AM, Christin A, Myers GD, Liu H, Cruz CR, et al (2009) Cytotoxic T lymphocyte therapy with donor T cells prevents and treats adenovirus and Epstein-Barr virus infections after haploidentical and matched unrelated stem cell transplantation. Blood 114: 4283–4292. 19700662 10.1182/blood-2009-07-232454 PMC2774556

[17] Leen AM, Myers GD, Sili U, Huls MH, Weiss H, et al (2006) Monoculture-derived T lymphocytes specific for multiple viruses expand and produce clinically relevant effects in immunocompromised individuals. Nat Med 12: 1160–1166. 16998485 10.1038/nm1475

[18] Feuchtinger T, Matthes-Martin S, Richard C, Lion T, Fuhrer M, et al (2006) Safe adoptive transfer of virus-specific T-cell immunity for the treatment of systemic adenovirus infection after allogeneic stem cell transplantation. Br J Haematol 134: 64–76. 16803570 10.1111/j.1365-2141.2006.06108.x

[19] Cobbold M, Khan N, Pourgheysari B, Tauro S, McDonald D, et al (2005) Adoptive transfer of cytomegalovirus-specific CTL to stem cell transplant patients after selection by HLA-peptide tetramers. J Exp Med 202: 379–386. 16061727 10.1084/jem.20040613 PMC2213070

[20] Feuchtinger T, Opherk K, Bethge WA, Topp MS, Schuster FR, et al (2010) Adoptive transfer of pp65-specific T cells for the treatment of chemorefractory cytomegalovirus disease or reactivation after haploidentical and matched unrelated stem cell transplantation. Blood 116: 4360–4367. 20625005 10.1182/blood-2010-01-262089

[21] Schmitt A, Tonn T, Busch DH, Grigoleit GU, Einsele H, et al (2011) Adoptive transfer and selective reconstitution of streptamer-selected cytomegalovirus-specific CD8+ T cells leads to virus clearance in patients after allogeneic peripheral blood stem cell transplantation. Transfusion 51: 591–599. 21133926 10.1111/j.1537-2995.2010.02940.x

[22] Uhlin M, Gertow J, Uzunel M, Okas M, Berglund S, et al.. (2012) Rapid Salvage Treatment With Virus-Specific T Cells for Therapy-Resistant Disease. Clin Infect Dis. 10.1093/cid/cis625 22806594

[23] Jones MS 2nd, Harrach B, Ganac RD, Gozum MM, Dela Cruz WP, et al (2007) New adenovirus species found in a patient presenting with gastroenteritis. J Virol 81: 5978–5984. 17360747 10.1128/JVI.02650-06 PMC1900323

[24] Leen AM, Sili U, Vanin EF, Jewell AM, Xie W, et al (2004) Conserved CTL epitopes on the adenovirus hexon protein expand subgroup cross-reactive and subgroup-specific CD8+ T cells. Blood 104: 2432–2440. 15265797 10.1182/blood-2004-02-0646

[25] Zandvliet ML, van Liempt E, Jedema I, Kruithof S, Kester MG, et al (2011) Simultaneous isolation of CD8(+) and CD4(+) T cells specific for multiple viruses for broad antiviral immune reconstitution after allogeneic stem cell transplantation. J Immunother 34: 307–319. 21389867 10.1097/CJI.0b013e318213cb90

[26] Zandvliet ML, Kester MG, van Liempt E, de Ru AH, van Veelen PA, et al (2012) Efficiency and Mechanism of Antigen-specific CD8+ T-cell Activation Using Synthetic Long Peptides. J Immunother 35: 142–153. 22306902 10.1097/CJI.0b013e318243f1ed

[27] Leen AM, Christin A, Khalil M, Weiss H, Gee AP, et al (2008) Identification of hexon-specific CD4 and CD8 T-cell epitopes for vaccine and immunotherapy. J Virol 82: 546–554. 17942545 10.1128/JVI.01689-07 PMC2224388

[28] Schipper RF, van Els CA, D'Amaro J, Oudshoorn M (1996) Minimal phenotype panels. A method for achieving maximum population coverage with a minimum of HLA antigens. Hum Immunol 51: 95–98. 8960911 10.1016/s0198-8859(96)00138-3

[29] Comoli P, Basso S, Labirio M, Baldanti F, Maccario R, et al (2008) T cell therapy of Epstein-Barr virus and adenovirus infections after hemopoietic stem cell transplant. Blood Cells Mol Dis 40: 68–70. 17904879 10.1016/j.bcmd.2007.06.020

[30] Sellar RS, Peggs KS (2012) The role of virus-specific adoptive T-cell therapy in hematopoietic transplantation. Cytotherapy 14: 391–400. 22420834 10.3109/14653249.2012.662769

[31] Gerdemann U, Keirnan JM, Katari UL, Yanagisawa R, Christin AS, et al (2012) Rapidly Generated Multivirus-specific Cytotoxic T Lymphocytes for the Prophylaxis and Treatment of Viral Infections. Mol Ther 20: 1622–1632. 22801446 10.1038/mt.2012.130 PMC3412490

[32] Garnett CT, Erdman D, Xu W, Gooding LR (2002) Prevalence and quantitation of species C adenovirus DNA in human mucosal lymphocytes. J Virol 76: 10608–10616. 12368303 10.1128/JVI.76.21.10608-10616.2002 PMC136639

[33] Rammensee H, Bachmann J, Emmerich NP, Bachor OA, Stevanovic S (1999) SYFPEITHI: database for MHC ligands and peptide motifs. Immunogenetics 50: 213–219. 10602881 10.1007/s002510050595

[34] Dohnal AM, Graffi S, Witt V, Eichstill C, Wagner D, et al (2009) Comparative evaluation of techniques for the manufacturing of dendritic cell-based cancer vaccines. J Cell Mol Med 13: 125–135. 18363835 10.1111/j.1582-4934.2008.00304.x PMC3823041

[35] Vellinga J, van den Wollenberg DJ, van der Heijdt S, Rabelink MJ, Hoeben RC (2005) The coiled-coil domain of the adenovirus type 5 protein IX is dispensable for capsid incorporation and thermostability. J Virol 79: 3206–3210. 15709043 10.1128/JVI.79.5.3206-3210.2005 PMC548437

[36] Ohrmalm L, Lindblom A, Omar H, Norbeck O, Gustafson I, et al (2011) Evaluation of a surveillance strategy for early detection of adenovirus by PCR of peripheral blood in hematopoietic SCT recipients: incidence and outcome. Bone Marrow Transplant 46: 267–272. 20400984 10.1038/bmt.2010.86

[37] Abate D, Cesaro S, Cofano S, Fiscon M, Saldan A, et al (2012) Diagnostic utility of human cytomegalovirus-specific T-cell response monitoring in predicting viremia in pediatric allogeneic stem-cell transplant patients. Transplantation 93: 536–542. 22314338 10.1097/TP.0b013e31824215db

[38] Gratama JW, Boeckh M, Nakamura R, Cornelissen JJ, Brooimans RA, et al (2010) Immune monitoring with iTAg MHC Tetramers for prediction of recurrent or persistent cytomegalovirus infection or disease in allogeneic hematopoietic stem cell transplant recipients: a prospective multicenter study. Blood 116: 1655–1662. 20508161 10.1182/blood-2010-03-273508

[39] Guerin-El Khourouj V, Dalle JH, Pedron B, Yakouben K, Bensoussan D, et al (2011) Quantitative and qualitative CD4 T cell immune responses related to adenovirus DNAemia in hematopoietic stem cell transplantation. Biol Blood Marrow Transplant 17: 476–485. 20869455 10.1016/j.bbmt.2010.09.010

[40] Olive M, Eisenlohr L, Flomenberg N, Hsu S, Flomenberg P (2002) The adenovirus capsid protein hexon contains a highly conserved human CD4+ T-cell epitope. Hum Gene Ther 13: 1167–1178. 12133270 10.1089/104303402320138952

[41] Serangeli C, Bicanic O, Scheible MH, Wernet D, Lang P, et al (2010) Ex vivo detection of adenovirus specific CD4+ T-cell responses to HLA-DR-epitopes of the Hexon protein show a contracted specificity of T(HELPER) cells following stem cell transplantation. Virology 397: 277–284. 19962170 10.1016/j.virol.2009.10.049

[42] Stemberger C, Huster KM, Koffler M, Anderl F, Schiemann M, et al (2007) A single naive CD8+ T cell precursor can develop into diverse effector and memory subsets. Immunity 27: 985–997. 18082432 10.1016/j.immuni.2007.10.012

[43] Boeckh M, Nichols WG (2004) The impact of cytomegalovirus serostatus of donor and recipient before hematopoietic stem cell transplantation in the era of antiviral prophylaxis and preemptive therapy. Blood 103: 2003–2008. 14644993 10.1182/blood-2003-10-3616

[44] Comoli P, Basso S, Zecca M, Pagliara D, Baldanti F, et al (2007) Preemptive therapy of EBV-related lymphoproliferative disease after pediatric haploidentical stem cell transplantation. Am J Transplant 7: 1648–1655. 17511690 10.1111/j.1600-6143.2007.01823.x

[45] Miller JS, Soignier Y, Panoskaltsis-Mortari A, McNearney SA, Yun GH, et al (2005) Successful adoptive transfer and in vivo expansion of human haploidentical NK cells in patients with cancer. Blood 105: 3051–3057. 15632206 10.1182/blood-2004-07-2974

[46] Linn YC, Niam M, Chu S, Choong A, Yong HX, et al (2012) The anti-tumour activity of allogeneic cytokine-induced killer cells in patients who relapse after allogeneic transplant for haematological malignancies. Bone Marrow Transplant 47: 957–966. 21986635 10.1038/bmt.2011.202

[47] Veltrop-Duits LA, Heemskerk B, Sombroek CC, van Vreeswijk T, Gubbels S, et al (2006) Human CD4+ T cells stimulated by conserved adenovirus 5 hexon peptides recognize cells infected with different species of human adenovirus. Eur J Immunol 36: 2410–2423. 16933360 10.1002/eji.200535786

[48] Chen BJ, Deoliveira D, Cui X, Le NT, Son J, et al (2007) Inability of memory T cells to induce graft-versus-host disease is a result of an abortive alloresponse. Blood 109: 3115–3123. 17148592 10.1182/blood-2006-04-016410 PMC1852216

[49] Melenhorst JJ, Leen AM, Bollard CM, Quigley MF, Price DA, et al (2010) Allogeneic virus-specific T cells with HLA alloreactivity do not produce GVHD in human subjects. Blood 116: 4700–4702. 20709906 10.1182/blood-2010-06-289991 PMC2996125

