# Supplementary material for: Short-Term In-Vitro Expansion Improves Monitoring and Allows Affordable Generation of Virus-Specific T-Cells against Several Viruses for a Broad Clinical Application
Source: PLoS One. 2013 Apr 22;8(4):e59592. doi: 10.1371/journal.pone.0059592 (PMC3632539; doi:10.1371/journal.pone.0059592)
Supplement: File S1 — Figure S1, Figure S2, Table S1 and Table S2. (DOC) [file pone.0059592.s001.doc]

**Figure S1. Frequency of EBV, and BKV-specific T-cells in donors before and after 6 and 12 day expansion analysed by multimers and the IFNg-CSA.** Percentage values of EBV-(A) and BKV-(B) specific streptamer^+^ T-cells among CD8^+^ from several donors, before (day0) and after 6 and 12 days of expansion, including representative dot plots. For some donors, streptamer analyses were only performed on days 0, 6 and/or 12. Six different HLA-dependent EBV-specific streptamers and one BKV-specific streptamer, were used, as indicated. Events of CD8^+^ and CD4^+^ IFN-γ secreting HAdV- (C) EBV-(D) and BKV-(E) specific T-cells analysed before (day 0) and after 6 and 12 days of expansion are shown. Filled symbols represent samples positive for HAdV-,EBV- or BKV-specific T cells, and open symbols represent samples under the limit of detection.


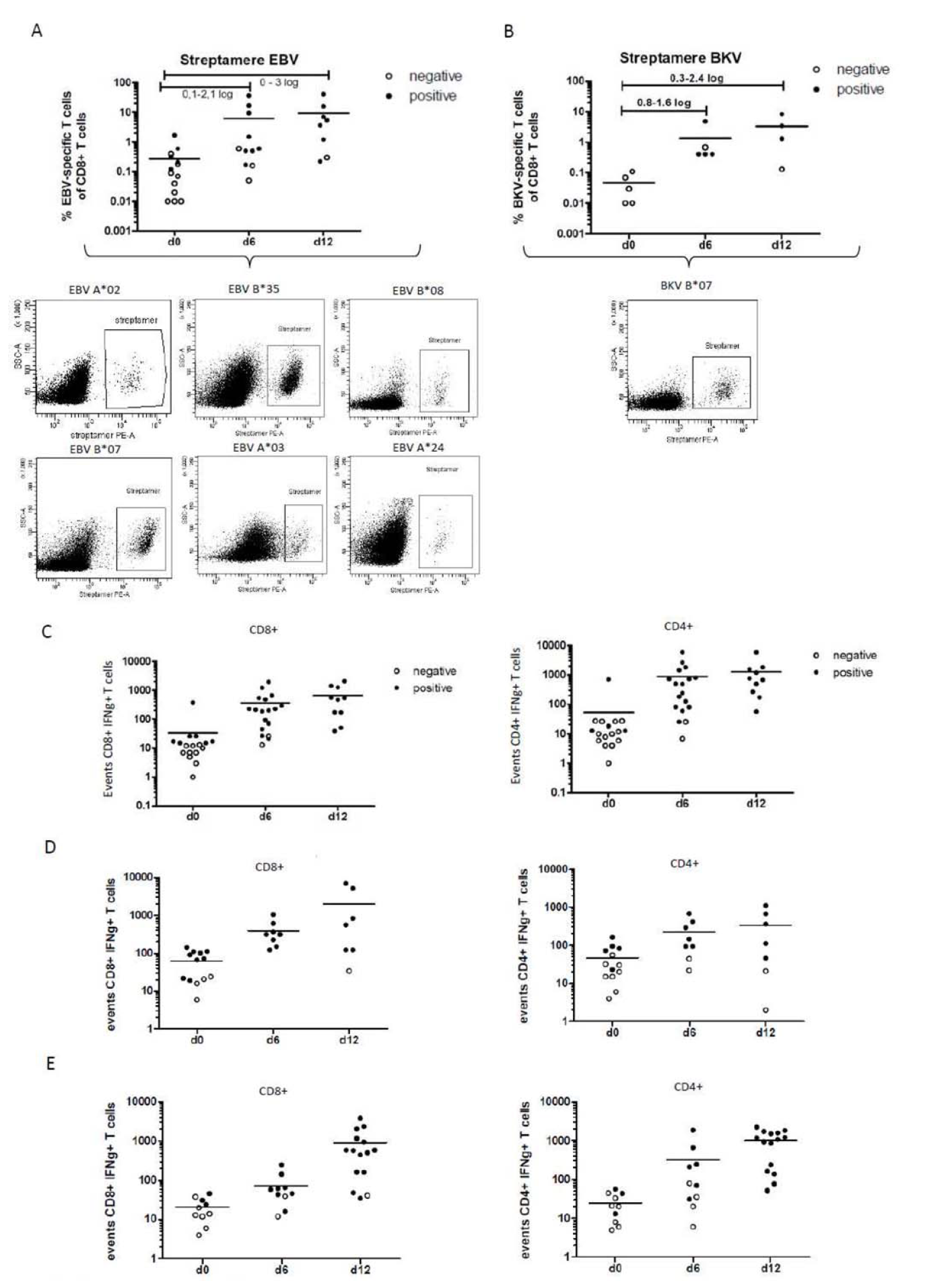


**Figure S2. Generation of functional short-term-expanded T-cells specific for EBV, CMV, and BKV.** Short-term expanded virus-specific T cells were generated using different peptide pools specific for CMV, EBV, and BKV. The absolute number of cultured cells, and of multimer^+^ T-cells, present per well at days 0 and 12 (A,D and G) are shown including mean +SEM. (B, E,and H). The percentage values of TCM, Naive, TEM and TEMRA T-cell populations within bulk CD8^+^ and CD4^+^ on days 0 and are shown. The graphs depict mean +SEM of 5 (CMV), 11 (EBV) and 7 (BKV). (C, F, and H) Specific lysis of autologous (auto) or mismatched allogeneic (allo) target cells unloaded (white bars) or loaded with the appropriate pepmix (black bars). The total number of “dying” target cells/µl was evaluated for each sample. Based on these, a summarizing graph of 3 to 7 different donors (as indicated) shows the percentage of dying target cells related to unloaded autologous (auto) or allogeneic (allo) target cells, which are set to 100%. Significance vs unloaded targets is shown in *p*-values as indicated: ns= not significant; *, p<=0.01;


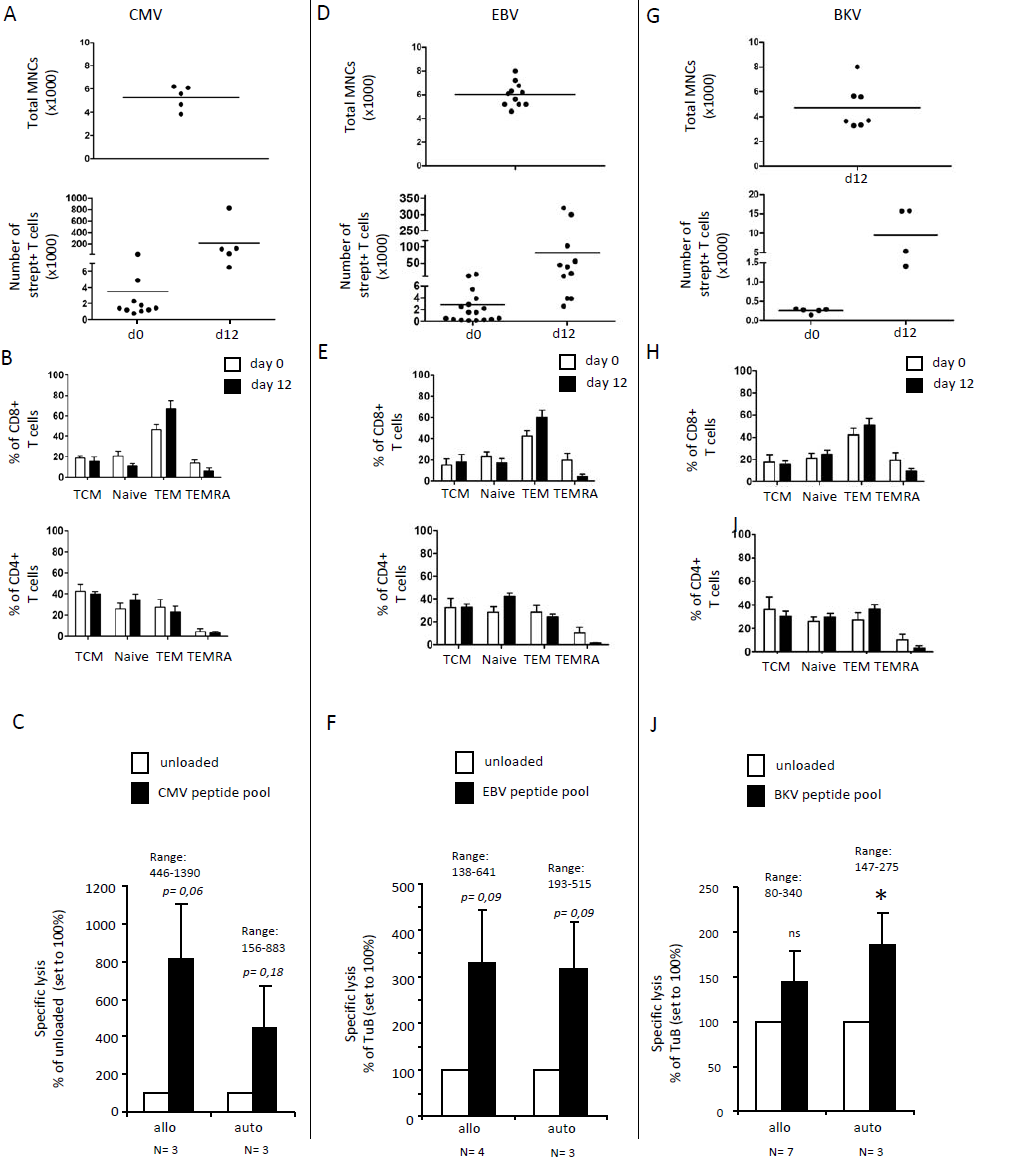


**Table S1. Peptides used in the ELIspot screening.**

Complete listing of peptides tested in the ELIspot screening with their sequences, SYFPEITHI score, proteins of origin and position in the ADV strains Ad2 and Ad5, and the number of responding and donors tested positive or negative for the predicted restricting allotype. Peptides are grouped according to their predicted or published restricting allotype. The following epitope sequences were published elsewhere, as indicated: TDLGQNLLY and LLYANSAHAL [49], TLLYVLFEV [40] and TFYLNHTFKK [24].

**HLA-A*01:**

| Sequence | SYFPEITHI  Score | Protein | Position  Ad2 | Position  Ad5 | Positive  A*01 | Positive  A*01 |
| --- | --- | --- | --- | --- | --- | --- |
| AQDYSTRINY | 27 | pVIII | 24 – 33  propeptide | 24 – 33  propeptide | 0 / 24 | n.a. |
| DIETPDTHISY | 23 | hexon | - | 291 – 301 | 1 / 40 | n.a. |
| DPDIMCSLCY | 28 | E1A | 166 – 175 | 166 – 175 | 0 / 24 | n.a. |
| EGEEFVLDY | 27 | E1A | 138 – 146 | 138 – 146 | 0 / 24 | n.a. |
| FIEEFVPSVY | 24 | pVIII | 77 – 86 | 77 – 86 | 0 / 24 | n.a. |
| FRDNFIGLMY | 28 | hexon | 340 – 349 | 328 – 337 | 6 / 42 | n.a. |
| GTEDELPNY | 29 | hexon | 416 – 424 | 404 – 412 | 1 / 24 | n.a. |
| IEEFVPSVY | 24 | pVIII | 78 – 86 | 78 – 86 | 0 / 24 | n.a. |
| ISDNPNTYDY | 30 | hexon | 509 – 516 | 493 – 502 | 0 / 24 | n.a. |
| LLDSIGDRTRY | 28 | hexon | 384 – 394 | 372 – 382 | 0 / 24 | n.a. |
| LQDRNTELSY | 28 | hexon | 372 – 381 | 360 – 369 | 3 / 40 | n.a. |
| LTDLGQNLLY | 36 | hexon | 900 – 909 | 884 – 893 | 68 / 73 | 0 / 16 |
| NAETQAKPVY | 25 | hexon | 196 – 205 | - | 0 / 24 | n.a. |
| PMDEPTLLY | 29 | hexon | 924 – 932 | 908 – 916 | 0 / 24 | n.a. |
| RSWPAALVY | 24 | pVIII | 72 – 80  propeptide | 72 – 80  propeptide | 0 / 24 | n.a. |
| TDLGQNLLY | 20 | hexon | 901 – 909 | 885 – 893 | 14 / 16 | 2 / 16 |
| TNDQSFNDY | 28 | hexon | 653 – 661 | 637 – 645 | 0 / 24 | n.a. |
| VLDRGPTFKPY | 26 | hexon | 105 – 115 | 105 – 115 | 0 / 24 | n.a. |
| VVDDTKYKDY | 26 | hexon | - | 804 - 813 | 0 / 24 | n.a. |
| VVDDTKYKEY | 27 | hexon | 820 – 829 | - | 0 / 24 | n.a. |
| YTYSGSIPY | 23 | hexon | 714 – 722 | 698 – 706 | 7 / 42 | 2 / 16 |

**HLA-A*02:**

| Sequence | SYFPEITHI  Score | Protein | Position  Ad2 | Position  Ad5 | Positive  A*02 | Positive  A*02 |
| --- | --- | --- | --- | --- | --- | --- |
| ALGPVSMPNL | 25 | E1A | 106 – 115 | 106 – 115 | 0 / 36 | n.a. |
| AVQEGIDLL | 24 | E1A | 73 – 81 | 73 – 81 | 0 / 36 | n.a. |
| FIEEFVPSV | 28 | pVIII | 77 – 85 | 77 – 85 | 1 / 36 | n.a. |
| FLCDRTLWRI | 24 | hexon | 879 – 888 | 863 – 872 | 5 / 36 | n.a. |
| FTPRQAILTL | 22 | pVIII | 51 – 60 | 51 – 60 | 2 / 28 | n.a. |
| HMISRVNGI | 24 | pVIII | 39 – 47  propeptide | 39 – 47  propeptide | 1 / 36 | n.a. |
| ILRRPTSPV | 25 | E1A | 213 – 221 | 213 – 221 | 0 / 38 | n.a. |
| ILVKQQNGKL | 24 | hexon | - | 248 – 257 | 1 / 36 | n.a. |
| LIGKTAVDSI | 24 | hexon | 865 – 874 | 849 – 858 | 0 / 36 | n.a. |
| LLDQLIEEV | 29 | E1A | 19 – 27 | 19 – 27 | 49 / 74 | 1 / 16 |
| LLNEPGQPL | 26 | E1A | - | 272 – 280 | 1 / 36 | n.a. |
| LLNESGQPL | 25 | E1A | 272 – 280 | - | 1 / 36 | n.a. |
| LLTPNEFEI | 23 | hexon | 752 – 760 | 736 – 744 | 0 / 28 | n.a. |
| LLYANSAHAL | 25 | hexon | 907 – 916 | 891 – 900 | 3 / 36 | 3 / 16 |
| MLLGNGRYV | 24 | hexon | 563 – 571 | 547 – 555 | 0 / 36 | n.a. |
| NLVPEVIDL | 26 | E1A | 114 – 122 | 114 – 122 | 0 / 36 | n.a. |
| QLAGGFRHRV | 25 | pVIII | 107 propeptide – 5 protein | 107 propeptide – 5 protein | 0 / 36 | n.a. |
| SAGPHMISRV | 22 | pVIII | 35 – 44  propeptide | 35 – 44  propeptide | 0 / 24 | n.a. |
| SLLDQLIEEV | 31 | E1A | 18 – 27 | 18 – 27 | 20 / 36 | n.a. |
| SMPNLVPEV | 28 | E1A | 111 – 119 | 111 – 119 | 18 / 61 | 6 / 16 |
| TFYLNHTFKK | 3 | hexon | 726 – 735 | 710 – 719 | 18 / 26 | 27 / 39 |
| TLAVGDNRV | 25 | hexon | 84 – 92 | 84 – 92 | 0 / 36 | n.a. |
| TLLYVLFEV | 27 | hexon | 929 – 937 | 913 – 921 | 38 / 57 | 6 / 12 |
| VINTETLTKV | 26 | hexon | - | 419 – 428 | 0 / 36 | n.a. |
| VLAGQASQL | 25 | hexon | 358 – 366 | 346 – 354 | 0 / 24 | n.a. |
| VLFEVFDVV | 24 | hexon | 933 – 941 | 917 – 925 | 7 / 56 | 0 / 16 |
| VLPRDAQAEV | 25 | pVIII | 90 – 99  propeptide | 90 – 99  propeptide | 4 / 36 | n.a. |
| YLNHTFKKV | 24 | hexon | 728 – 736 | 712 – 720 | 2 / 36 | n.a. |
| YVLFEVFDVV | 17 | hexon | 932 – 941 | 916 – 925 | 14 / 29 | 0 / 16 |

**HLA-A*24:**

| Sequence | SYFPEITHI  Score | Protein | Position  Ad2 | Position  Ad5 | Positive  A*24 | Positive  A*24 |
| --- | --- | --- | --- | --- | --- | --- |
| AYPANFPYPL | 23 | hexon | 856 – 865 | 840 – 849 | 14 / 40 | 0 / 16 |
| AYSYKARFTL | 21 | hexon | 76 – 85 | 76 – 85 | 1 / 16 | n.a. |
| CYMRTCGMF | 20 | E1A | 174 – 182 | 174 – 182 | 2 / 16 | n.a. |
| DYMDNVNPF | 22 | hexon | 542 – 550 | 526 – 534 | 0 / 16 | n.a. |
| DYLSAANML | 22 | hexon | 660 – 668 | 644 – 652 | 1 / 16 | n.a. |
| EYLSPGLVQF | 22 | hexon | 21 – 30 | 21 – 30 | 0 / 16 | n.a. |
| HYPDQFIPNF | 24 | pVIII | 96 – 105 | 96 – 105 | 0 / 16 | n.a. |
| KYKDYQQVGI | 21 | hexon | - | 809 – 818 | 1 / 16 | n.a. |
| KYKEYQQVGI | 22 | hexon | 825 – 834 | - | 0 / 16 | n.a. |
| KYNPTNVEI | 22 | hexon | 501 – 509 | - | 1 / 16 | n.a. |
| LYSNIALYL | 21 | hexon | 488 – 496 | 472 – 480 | 0 / 16 | n.a. |
| NYIAFRDNF | 22 | hexon | 336 – 344 | 324 – 332 | 1 / 16 | n.a. |
| NYIAFRDNFI | 22 | hexon | 336 – 345 | 324 – 333 | 12 / 38 | 9 / 22 |
| NYMSAGPHMI | 21 | pVIII | 32 – 41  propeptide | 32 – 41  propeptide | 2 / 16 | n.a. |
| PYLDGTFYL | 24 | hexon | 721 – 729 | 705 – 713 | 0 / 16 | n.a. |
| SFTPRQAIL | 18 | pVIII | 50 – 58 | 50 – 58 | 1 / 16 | n.a. |
| SYDPDVRII | 22 | hexon | 404 – 412 | 393 – 401 | 0 / 16 | n.a. |
| SYKDRMYSF | 21 | hexon | 801 – 809 | 785 – 793 | 0 / 16 | n.a. |
| TYFDIRGVL | 23 | hexon | 98 – 106 | 98 – 106 | 1 / 16 | n.a. |
| TYFSLNNKF | 23 | hexon | 36 – 44 | 36 – 44 | 44 / 58 | 1 / 22 |
| TYSGSIPYL | 22 | hexon | 715 – 723 | 699 – 707 | 0 / 16 | n.a. |

**Table S2**. Type of peptide-sequences used according to their MHC class I type

|  |  |  |  |  |  |
| --- | --- | --- | --- | --- | --- |
| Virus | Protein | Peptide | HLA-Type | Streptamers (IBA) | Pentamers (Proimmne) |
| ADV | Hexon AdV2 | LTDLGQNLLY | A*01:01 | yes | no |
|  | Hexon AdV5 | TDLGQNNLY | A*01:01 | no | yes |
|  |  | TDLGQNMLY | A*01:01 | no | no |
|  |  | TYFSLNNKF | A*24:02 | yes | yes |
|  |  | TYFTLGNKF | A*24:02 | no | no |
|  |  | TYFFLGNKF | A*24:02 | no | no |
|  |  | KPYSGTAYNAL | B*07:02 | yes | yes |
|  |  | KPYSGTAYNSL | B*07:02 | no | no |
|  |  | MPNRPNYIAF | B*35:01 | yes | yes |
|  |  | MPNRPNYIGF | B*35:01 | no | no |
|  |  | APNRANYIAF | B*35:01 | no | no |
|  |  | APNRPNYIGF | B*35:01 | no | no |
|  | E1A | LLDQLIEEV | A*02:01 | yes | no |
| CMV | pp50 | VTEHDTLLY | A*01:01 | yes | no |
| EBV | EBNA-3A | RLRAEAQVK | A*03:01 | yes | no |
|  |  | RPPIFIRRL | B*07:02 | yes | no |
|  |  | FLRGRAYGL | B*08:01 | yes | no |
|  | LMP-2 | FLYALALLL | A*02:01 | yes | no |
|  |  | PYLFWLAAI | A*24:02 | yes | no |
|  | EBNA-1A | HPVGEADYFEY | B*35:01 | yes | no |
| BKV | LT-Ag | RLDSEISMY | A*01:01 | yes | no |
|  |  | LLLIWFRPV | A*02:01 | yes | no |
|  |  | PYHTIEESI | A*24:02 | yes | no |
|  |  | LPLMRKAYL | B*07:02 | yes | no |
| IBA Technologies (Göttingen, Germany) | | |  |  |  |
| Proimmune (Oxford, UK) | |  |  |  |  |
